# Lead-Free Double Cs_2_Ag(Bi,Sb)(Br,I)_6_ Perovskites: Going below 1.8 eV Bandgap by Anion Exchange and Solid-State Reactions

**DOI:** 10.1021/jacs.6c07190

**Published:** 2026-05-18

**Authors:** Oleksandr Stroyuk, Oleksandra Raievska, Sachin Kinge, Jens Hauch, Christoph J. Brabec

**Affiliations:** † Institute of Energy Materials and Devices - Photovoltaics (IMD-3), 28334 Forschungszentrum Jülich GmbH, Erlangen 91058, Germany; ‡ Materials Engineering Div., Toyota Motors Europe, Brussels 1140, Belgium; § Materials for Electronics and Energy Technology (i-MEET), Friedrich-Alexander-Universität Erlangen-Nürnberg, Erlangen 91058, Germany

## Abstract

Anion exchange of microcrystalline Cs_2_AgBiBr_6_ double perovskite (CAB-B) with NaI under ambient conditions yielded mixtures of Cs_3_Bi_2_(Br,I)_9_ (CB-(B)­I) and CsAg_2_I_3_ (CA-I) double salts, which were transformed by annealing at 250–300 °C into a tetragonal Cs_2_AgBi­(Br,I)_6_ double perovskite (CAB-(B)­I) with ca. 80 mol % iodide. The thermally activated solid-state reaction between CB-(B)I and CA-I was confirmed by annealing mechanically mixed CB-(B)I and CA-I, which yielded CAB-(B)I perovskites. Optimization of the anion exchange and the solid-state reaction (AE/T) resulted in phase-pure CAB-(B)I perovskite with ca. 90 mol% iodide and a band gap slightly below 1.9 eV. The general applicability of the proposed approach was demonstrated in a series of AE/T-driven transformations of more complex precursors, including the conversion of Cs_2_AgBi_*x*_Sb_1–*x*_Br_6_ into tetragonal Cs_2_AgBi_*x*_Sb_1–*x*_(Br,I)_6_ perovskites with variable *x*, the highest iodide content of ca. 90%, and the lowest band gap of 1.78 eV observed at a Bi/Sb ratio of 1:1, as well as solid-state reactions between ternary mixtures of CB-(B)­I, CA-I, and Cs_3_Bi_2_Br_9_ double salts, yielding tetragonal Cs_2_AgBi­(Br_*y*_I_1–*y*_) perovskites with linear compositional variations of the lattice parameters and band gaps over a broad range of *y* = 0.08–0.76. The reported two-stage AE/T approach is highlighted as a general, flexible, and sustainable pathway for the combinatorial synthesis of stable tetragonal double perovskites with variable compositions and levels of complexity.

## Graphical abstract

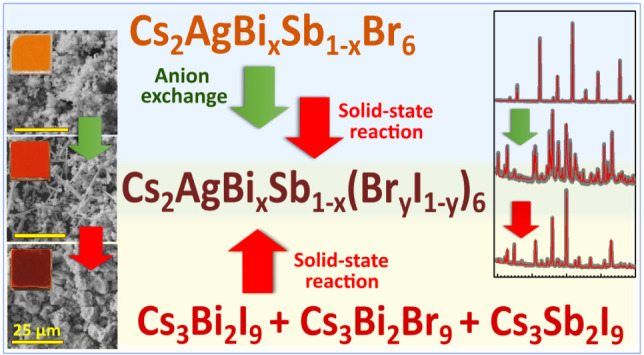

## 1 Introduction

Lead-free double halide perovskites or elpasolites with a general structure A^I^_2_M^I^M^III^X_6_ (A^I^, M^I^, M^III^—metal cations, X—halide anions) feature a prominent compositional diversity with four crystallographic positions available for independent variations, which is further enhanced by a high structural tolerance to dopings and multiple alloyings on each of the four positions.^1–7^ The unique lattice composed of an infinite octahedral network and the compositional variability of double perovskites offer significant potential for multiple optoelectronic applications, including photovoltaics (PV), photodetection, light emission, photocatalysis, sensor devices, and nonvolatile memory elements.^1–13^

One of the key halide elpasolite compounds tested for numerous applications is Cs_2_AgBiBr_6_, which is often targeted for its favorable combination of high stability, availability, visible-light sensitivity, and solution processability.^1,4,5,7,12–14^ Many applications of Cs_2_AgBiBr_6_ perovskite, particularly in PV, are strongly limited by its relatively large bandgap of approximately 2.2 eV, prompting attempts to partially or completely substitute bromide anions with iodide while preserving the perovskite structure. The product of complete anion exchange, Cs_2_AgBiI_6_, was theoretically predicted to have a band gap of 1.6 eV and a density of states 3 orders of magnitude higher than Cs_2_AgBiBr_6_,^15^ while having a much lower exciton binding energy and more delocalized charge carriers,^16^ both promising for PV applications. An experimental indirect band gap of 1.75 eV was recently reported by D. Gamelin’s group for Cs_2_AgBiI_6_ nanocrystals (NCs) produced by anion exchange.^17^ However, until recently, attempts to synthesize stable microcrystalline Cs_2_AgBiI_6_ perovskite, as well as other iodide double perovskites, were unsuccessful, even for compounds theoretically predicted to be relatively stable.^18–22^ A significant potential for the synthesis of double (bromo)­iodide perovskites was recognized through mechanochemical treatments of complex precursors;^23^ however, so far, only bromoiodide perovskites with a low iodide content (ca. 3%) have been prepared by ball milling as single-phase products.^24^

In 2023, Gamelin’s group reported probably the first experimental evidence of the synthesis of stable Cs_2_AgBiI_6_ perovskite in the form of ligand-stabilized NCs derived from Cs_2_AgBiBr_6_ NCs by anion exchange.^25^ A detailed study of Cs_2_AgBiI_6_ NCs by synchrotron X-ray diffraction revealed their tetragonal (space group *I*4̅*m*) structure, rather than cubic *Fm*3̅*m* symmetry, typical for double perovskites. The microcrystalline Cs_2_AgBiI_6_ produced by a similar approach was unstable and decomposed into simpler compounds, indicating that the stability of 10–15 nm Cs_2_AgBiI_6_ NCs is provided by specific surface ligands.^25^ Stabilizing larger/or ligand-free Cs_2_AgBiI_6_ crystals so far remains a challenge.

The approach of anion-exchange-driven conversion of more stable chloride or bromide perovskites into iodide derivatives is highly promising, as it avoids using unstable/volatile iodide compounds, can be total or partial, and performed in any direction, for example, converting bromides into corresponding lower-bandgap iodides or higher-bandgap chlorides.^5,17,25,26^ In 2025, D. Gamelin’s group reported a successful anion-exchange-based synthesis of ligand-capped Cs_2_AgSbI_6_ perovskite NCs, retaining stability in air up to 140 °C, though predicted as unstable by *ab initio* calculations.^26^

Recently, we have explored the versatility of mild and open-atmosphere anion-exchange of a series of lead-free Cs_2_AgM^III^Cl_6_ perovskites^27^ and perovskite-like Cs_3_M^III^_2_X_9_ double salts (X = Cl, Br)^28^ using stable and benign NaBr and NaI as sources of bromide and iodide. Typically, thermodynamically stable single-phase products precipitated at room temperature contained two (Cl + Br, Br + I) or three halide anions (Cl + Br + I), indicating that their stability can, at least partially, be provided by halide alloying. For example, very small amounts of bromide (5–10 mol %) perfectly stabilize Cs_3_Bi­(Cl,Br,I)_9_ double salts, whereas less complex Cs_3_Bi_2_(Cl,I)_9_ compounds cannot be isolated as stable products.^28^ Small bromide additions were also found to stabilize trigonal Cs_3_Bi­(Cl,Br,I)_9_ compounds, while purely iodide double salts crystallize in a hexagonal lattice.^28^

These examples indicate that halide mixing can stabilize certain phases, lattice symmetries, and compositions that are not possible under the given conditions for counterparts with individual or simpler halide components. At that, the compositional tailoring of the halide component can be expected to exert a stabilizing effect on unstable elpasolite compounds, similar to the surface ligand effect observed for Cs_2_AgBiI_6_ NCs.^25^ In this view, the present work aims at the exploration of the feasibility of the formation of stable microcrystalline bromo-iodide Cs_2_Ag­(Bi,Sb)­(Br,I)_6_ elpasolites by mild anion-exchange transformations of double Cs_2_Ag­(Bi,Sb)­Br_6_ perovskites combined with the postexchange thermal activation of the intermediate products.

We start by introducing a modified approach to the synthesis of Cs_2_AgBiBr_6_ perovskite, discuss the composition and properties of the intermediate products formed during its anion exchange with sodium iodide, and their conversion into tetragonal bromoiodide Cs_2_AgBi­(Br,I)_6_ perovskites via thermally activated solid-state reactions between the intermediates (Section 1). Then, we provide experimental evidence for the feasibility of such solid-state reactions by synthesizing Cs_2_AgBi­(Br,I)_6_ perovskites from mechanically mixed separate double salts (Section 2); perform the optimization of the two-stage anion exchange/annealing conversion of Cs_2_AgBiBr_6_ to achieve the highest yields of Cs_2_AgBi­(Br,I)_6_ perovskites with the lowest band gaps; and show the feasibility of producing phase-pure products by an additional grinding/annealing step (Section 3). Finally, the general character of the proposed two-stage approach is demonstrated by applying it to more complex anion-exchange precursors and to more complex mixtures of halide double salts introduced into thermal solid-state reactions (Section 4).

## 2 Results and Discussion

### 2.1 Transformations of Cs_2_AgBiBr_6_ Perovskite Driven by Anion Exchange and Annealing

#### 2.1.1 Synthesis of Cs_2_AgBiBr_6_ Perovskite Revisited

Typically, double Cs_2_AgBiBr_6_ perovskite is produced by dissolving individual metal bromides—CsBr, AgBr, and BiBr_3_—in concentrated (48 wt %) HBr at 110 °C, followed by the precipitation of microcrystalline Cs_2_AgBiBr_6_ during gradual cooling of the solution.^4,5,7^ This approach, though popular, has at least two substantial drawbacks. First, it requires prolonged heating (approximately 2 h) of the precursors in an aggressive HBr medium to dissolve CsAgBr_2_ perovskite, a low-solubility byproduct. Second, silver bromide is highly sensitive to ambient light and requires shielding of the precursor and reactor from daylight.

Here, we develop an alternative approach that yields Cs_2_AgBiBr_6_ as a single-phase product under open-air conditions at room temperature (RT) and at a much lower hydrobromic acid concentration. Similar to our recent nomenclature for perovskite-like double salts,^28^ we denote halide compounds by the first letters of the elements, separating the cations and anions with a hyphen. In this scheme, Cs_2_AgBiBr_6_ perovskite will be further denoted as CAB-B. The complete list of abbreviations used to refer to halide compounds is presented in the Electronic Supporting Information (ESI).

The revised synthesis of CAB-B perovskite follows our previous protocols for the synthesis of double chloride perovskites,^27,29,30^ with a key step being the separation of M^+^ and M^3+^ cations in two precursor solutions. The first precursor contains BiBr_3_ dissolved in diluted HBr; the second comprises cesium acetate and silver nitrate in a water/2-propanol mixture that acts as an antisolvent, precipitating CAB-B perovskite immediately upon mixing the two precursors. In this scheme, no AgBr is used, and the formation of low-soluble CsAgBr_2_ is avoided, enabling RT synthesis with a minimal amount of HBr. Both precursors are stable: Bi^3+^ is protected from hydrolytic processes by an excess of HBr, and Ag^+^ is bound in a complex with ammonia to avoid hydrolysis in the slightly alkaline environment produced by cesium acetate. The synthesis is scalable to multigram quantities of single-phase cubic CAB-B and is adaptable to more complex compositions, particularly Cs_2_AgBi_*x*_Sb_1–*x*_Br_6_ (CABS-B) perovskites, discussed in Section 4. Details of all synthetic procedures and characterizations are presented in the ESI. The CAB-B was characterized by scanning electron microscopy (SEM), energy-dispersive X-ray spectroscopy (EDX), powder X-ray diffraction (PXRD), and UV–vis absorption spectroscopy.

The SEM inspection showed CAB-B as loosely aggregated microcrystals with a broad grain size distribution of ca. 0.2–2.0 μm (Figure 1a). The composition and stoichiometry of CAB-B identified by EDX analysis averaged from at least four different measurements (see more details in ESI and exemplary Figure S1) are typical for double perovskites with Br/Bi, Bi/Ag, and Cs/Bi ratios of 6.0, 1.0, and ca. 2, respectively (Table 1, sample ID CAB-B). A Rietveld refinement of the PXRD pattern of CAB-B (Figure 1b, curve 1) showed the presence of a single cubic phase belonging to the *Fm*3̅*m* space symmetry group and lattice parameter *L* = 11.282(2) Å, both typical for the CAB-B perovskite.^4,5,7^

**Table 1:** Composition of Selected Samples

		X = Br + I							
	**Sample ID**	Br, %	I, %	**X/M** ^**III**^	X/Ag	**Cs/M** ^**III**^	Cs/Ag	**Bi/M** ^**III**^	**Formal composition**
CAB-B		100	0	6.0	6.0	2.2	1.9	1.0	Cs_2_AgBiBr_6_
AE-CAB-B	Site #1	20	80	4.6	-	1.5	-	1.0	Cs_3_Bi_2_(Br_0.20_I_0.80_)_9_
	Site #2	5	95	-	1.4	-	0.6	-	CsAg_2_I_3_
CAB-(B)I, 250 °C		20	80	6.1	6.1	2.0	2.0	1.0	Cs_2_AgBi(Br_0.2_I_0.8_)_6_
CAB-(B)I, 300 °C		12	88	6.1	5.7	2.1	2.0	1.0	Cs_2_AgBi(Br_0.12_I_0.88_)_6_
CABS-B, nom. Bi:Sb = 1:1		100	0	5.7	5.6	2.1	2.0	0.48	Cs_2_Ag(Bi_0.5_Sb_0.5_)Br_6_
AE-CABS-B	Site #1	22	78	4.6	-	1.5	-	0.52	Cs_3_(Bi_0.5_Sb_0.5_)_2_(Br_0.22_I_0.78_)_9_
	Site #2	3	97	-	1.5	-	0.6	-	CsAg_2_I_3_
CABS-(B)I, nom. Bi:Sb = 1:1, 300 °C		12	88	5.8	5.9	1.6	1.8	0.49	Cs_2_Ag(Bi_0.5_Sb_0.5_)(Br_0.12_I_0.88_)_6_

**Figure 1:**
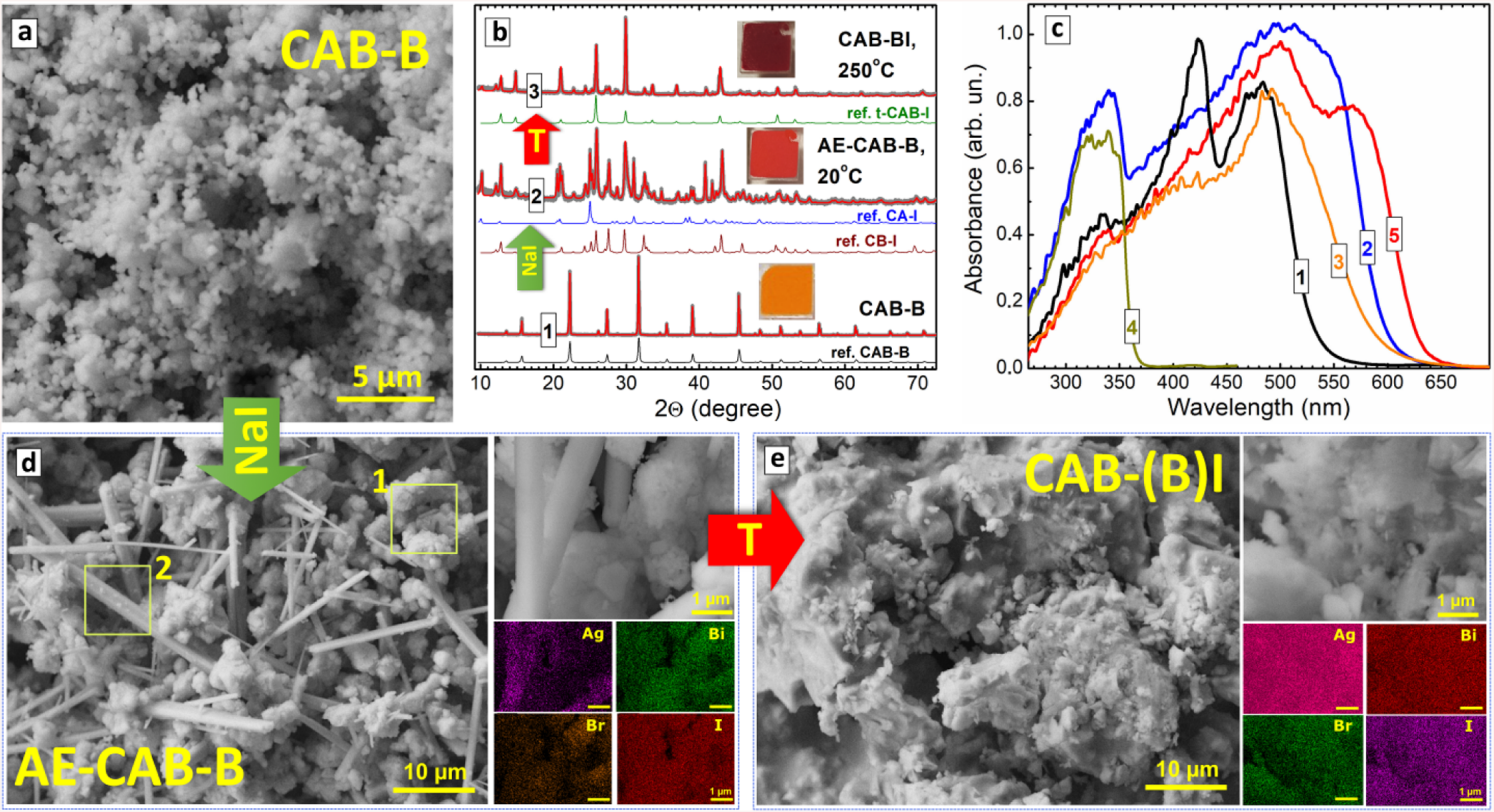
(a) SEM image of CAB-B perovskite; (b, c) Powder XRD patterns (b) and absorption spectra (c) of CAB-B (curves 1), products of the anion exchange with NaI at 20 °C (2), and subsequent annealing at 250 °C (5). In (b), the gray and red lines show experimental data and Rietveld refinement, respectively; inserts show photographs of corresponding samples drop-cast on glass. In (c), curves (3) and (4) show absorption spectra of Cs_3_Bi_2_I_9_ and CsAg_2_I_3_, respectively. (d, e) SEM images and element distribution maps for Ag, Bi, Br, and I for the products of anion exchange of CAB-B (AE-CAB-B) with NaI at 20 °C (d) and their thermal conversion into CAB-(B)I perovskite by the open-air annealing at 250 °C (e). In (d), rectangles (1) and (2) mark two distinct morphologies selected for EDX analysis (see Table 1).

The absorption spectrum of CAB-B (Figure 1c, curve 1) revealed a continuous absorption band with an edge at 530 nm and three distinct peaks typically assigned to specific localized electron transitions in BiBr_6_ and AgBr_6_ octahedra.^4,14^ The representation of the absorption spectrum of CAB-B in Tauc coordinates for both indirect and direct allowed electronic transitions yields reasonably long linear sections (ESI, Figure S2a), allowing accurate estimation of the bandgap as 2.28 eV (indirect transition) and 2.41 eV (direct transition), the values being typical for CAB-B perovskite.^4,17^ The ambiguity in assigning the transition type responsible for the absorption band edge was reported experimentally^4,14^ and also inferred from *ab initio* calculations.^31^

### 2.2 Anion-Exchange-Driven Conversion of CAB-B

Recently, we have reported a mild open-environment conversion of Cs_3_(Bi_*x*_Sb_1–*x*_)_2_Br_9_ double salts into corresponding bromo-iodide compounds by anion exchange (AE) using NaI as a stable and benign iodide source.^28^ Here, this approach is extended to CAB-B perovskite (see the ESI for details).

The interaction between CAB-B and NaI at RT results in a shift of the absorption band edge from 530 nm to ca. 600 nm (Figure 1c, curve 2, see also photographs in inserts), indicating the transformation of CAB-B into an iodide derivative with a lower bandgap. Inspection of the AE product by PXRD showed it to be a mixture of two phases (Table 1, sample ID AE-CAB-B), Cs_3_Bi_2_I_9_ double salt (ca. 80 wt %) and CsAg_2_I_3_ double salt (Figure 1b, curve 2), rather than the expected iodide double perovskite.

Following PXRD data, SEM inspection revealed two distinct morphologies in the anion-exchanged sample: aggregated polygonal microcrystals and microrods with a length of 10–20 μm (Figure 1d). An EDX analysis of two selected areas, corresponding to microcrystals and microrods (marked by rectangles 1 and 2 in Figure 1d; additional exemplary EDX data are provided in ESI, Figure S3), indeed showed the microcrystals to be Cs_3_Bi_2_(Br,I)_9_ double salts with 20 at. % of residual bromide, enriched by bismuth and bromide (see elemental maps in Figure 1d). This compound is further referred to as CB-(B)­I, the parentheses denoting the inadvertent presence of residual bromine. The microrods were found to be enriched in silver and iodide and were identified as CsAg_2_I_3_ (CA-I), consistent with the PXRD results.

In line with the PXRD and EDX data, the absorption spectrum of the AE-CAB-B is close to the sum of the absorption spectra of individual CB-I (Figure 1c, curve 3) and CA-I (Figure 1c, curve 4), as discussed in more detail in the next section. At that, CA-I introduces a distinct absorption peak at 320–340 nm, while CB-I contributes with a peak at 480–490 nm and a band edge at ca. 590 nm.

### 2.3 Thermally Activated Conversion of AE-CAB-B

The first experimental evidence of the formation of a tetragonal modification of Cs_2_AgBiI_6_ (CAB-I) double perovskite nanocrystals (NCs) via anion exchange in CAB-B NCs was reported by Gamelin’s group.^25^ The stability of CAB-I NCs was attributed to the effect of surface stabilization by ligands, while similar transformations of microcrystalline CAB-B resulted in CAB-I decomposition described by the following reaction:

1 $$2{\mathrm{Cs}}_{2}{\mathrm{AgBiI}}_{6}\to {\mathrm{Cs}}_{3}{\mathrm{Bi}}_{2}I_{9}+\mathrm{CsI}\;+2\mathrm{AgI}$$

Considering that the anion-exchange-driven transformation of CAB-B observed here yields very similar mixtures of CB-(B)I and CA-I, we attempted to reverse reaction (1) back to the formation of CAB-I or a bromo-iodide Cs_2_AgBi­(Br,I)_6_ perovskite by thermal activation of the anion-exchange products through annealing.

The original CAB-B perovskite was found to be thermally stable, showing no morphological, structural, or spectral changes after annealing at 250 °C in air (ESI, Figure S4). In contrast, the annealing of the as-prepared AE-CAB-B, that is, the mixture of CB-(B)I and CA-I, results in a further shift of the absorption band edge to ca. 630 nm (Figure 1c, curve 5), with the color of the sample permanently changing from light-brown to dark-red-brown (see photographs in Figure 1b). At that, the absorption peak of CA-I disappears almost completely, both observations indicating that chemical transformations take place in the system.

This conclusion is corroborated by a considerable increase in the level of symmetry shown by the PXRD pattern of the annealed product as compared to AE-CAB-B (Figure 1b, curve 3). Using the structural parameters of tetragonal CAB-I reported by Gamelin’s group,^23^ in particular, *I*4̅*m* space group and lattice parameters *a* = *b* = 8.535(4) Å, *c* = 12.080(4) Å, and an elementary cell volume of *V* = 880 Å^3^, as a starting input for the Rietveld refinement, we identified the dominating phase in the annealing products as a tetragonal perovskite with *a* = *b* = 8.422(1) Å and *c* = 11.911(2) Å, corresponding to *V* = 845 Å^3^. The lower cell parameter values, compared with the reported CAB-I perovskite, were attributed to residual bromide, as further confirmed by EDX. The best fit with the experimental PXRD pattern was achieved for a combination of three phases, comprising 60 wt.% tetragonal CAB-BI perovskite, 30 wt.% CB-(B)­I, and 10 wt.% CA-I, indicating an incomplete character to the thermally activated solid-state reaction between CB-(B)I and CA-I at a given annealing temperature of 250 °C.

Examination of the annealed product with SEM revealed a significant change in the morphology of the anion-exchanged products upon annealing (Figure 1e). The original mixture of microcrystals and microrods was transformed into much larger, denser formations, giving the visual impression of melting within the system during thermal treatment. The large-area EDX analysis of the product revealed stoichiometry typical for a double perovskite, with X/Bi, Cs/Bi, and Cs/Ag ratios of ca. 6, 2, and 2, respectively (Table 1; ESI, Figure S5). The halide component comprised 80% iodide and 20% bromide, in line with the above-discussed PXRD observations.

In summary, the dominating phase in the annealed product was identified as a tetragonal double perovskite with a bruto composition of Cs_2_AgBi­(Br_0.2_I_0.8_)_6_. In further discussion, the bromo-iodide perovskites produced via sequential anion exchange and annealing will be denoted as CAB-(B)­I, with the parentheses referring to the residual character of bromide content and to distinguish these compounds from Cs_2_AgBi­(Br_*y*_I_1–*y*_)_6_ with freely variable bromide fraction, discussed in Section 4. Spatial mapping of element distribution by EDX showed a uniform distribution of all components, with no noticeable differences in Br- and I-related maps (Figure 1e), indicating homogeneous alloying of bromide and iodide anions in the perovskite lattice.

Despite the multiphase character of the product annealed at 250 °C, with only 60% being tetragonal CAB-(B)I perovskite, the bandgap of CAB-(B)I still can be evaluated from the absorption spectrum, while neither CB-(B)I nor CA-I contributes in this spectral range. Similar to CAB-B perovskite, the absorption spectrum of CAB-(B)I shows extended linear sections in Tauc coordinates for both indirect and direct allowed electronic transitions (ESI, Figure S2b), providing no clear distinctions between the two transition types. The indirect and direct band gaps were evaluated as 1.91 and 2.03 eV, respectively.

## 3 Proving the Feasibility of the Solid-State Reaction between CB-(B)I and CA-I

Direct evidence of the solid-state reaction between CB-(B)I and CA-I, resulting in the formation of tetragonal CAB-(B)I perovskite, was collected by observing annealing-induced transformations in mechanical mixtures of separately produced CB-(B)I and CA-I double salts, as well as by deeper chemical transformations of AE-CAB-B annealed with additional CA-I.

### 3.1 Solid-State Reaction between CB-(B)I and CA-I

The CB-(B)I double salt was synthesized by previously reported anion exchange from CB-B and contains ca. 10 at. % residual bromide.^28^ The CA-I double salt was prepared by direct interaction of the above-discussed Cs + Ag precursor with NaI solution in water/2-propanol (the CA-I synthesis was developed specifically for these experiments; see details in the ESI). The CB-(B)I and CA-I were mechanically mixed at RT, converted into suspensions in 2-propanol, drop-cast as films on glass substrates, and subjected to annealing at 250 °C in the open air environment.

The structure and phase purity of CB-(B)I and CA-I were verified by the Rietveld refinement of corresponding PXRD patterns (Figure 2a). No trace of CAB-(B)­I perovskite was detected in PXRD profiles of the mechanical mixture of CB-(B) + CA-I kept at RT, regardless of the observation period (up to several weeks). However, even a relatively short thermal exposure of the mixture at 250 °C results in the formation of the tetragonal CAB-(B)I phase, amounting to 50 wt.% for 2 min annealing and to 70% for 5 min annealing (Figure 2a). At that, no phase transitions and reactions were observed for the individual CB-(B)I as well as for the mixtures of CB-(B)­I with AgI annealed in the same conditions (ESI, Figure S6). The latter observation indicates a selective character of the solid-state reaction—it requires a specific combination of CB-(B)I and CA-I phases, not merely the simultaneous presence of all constituent elements, to yield tetragonal CAB-(B)I perovskite.

**Figure 2:**
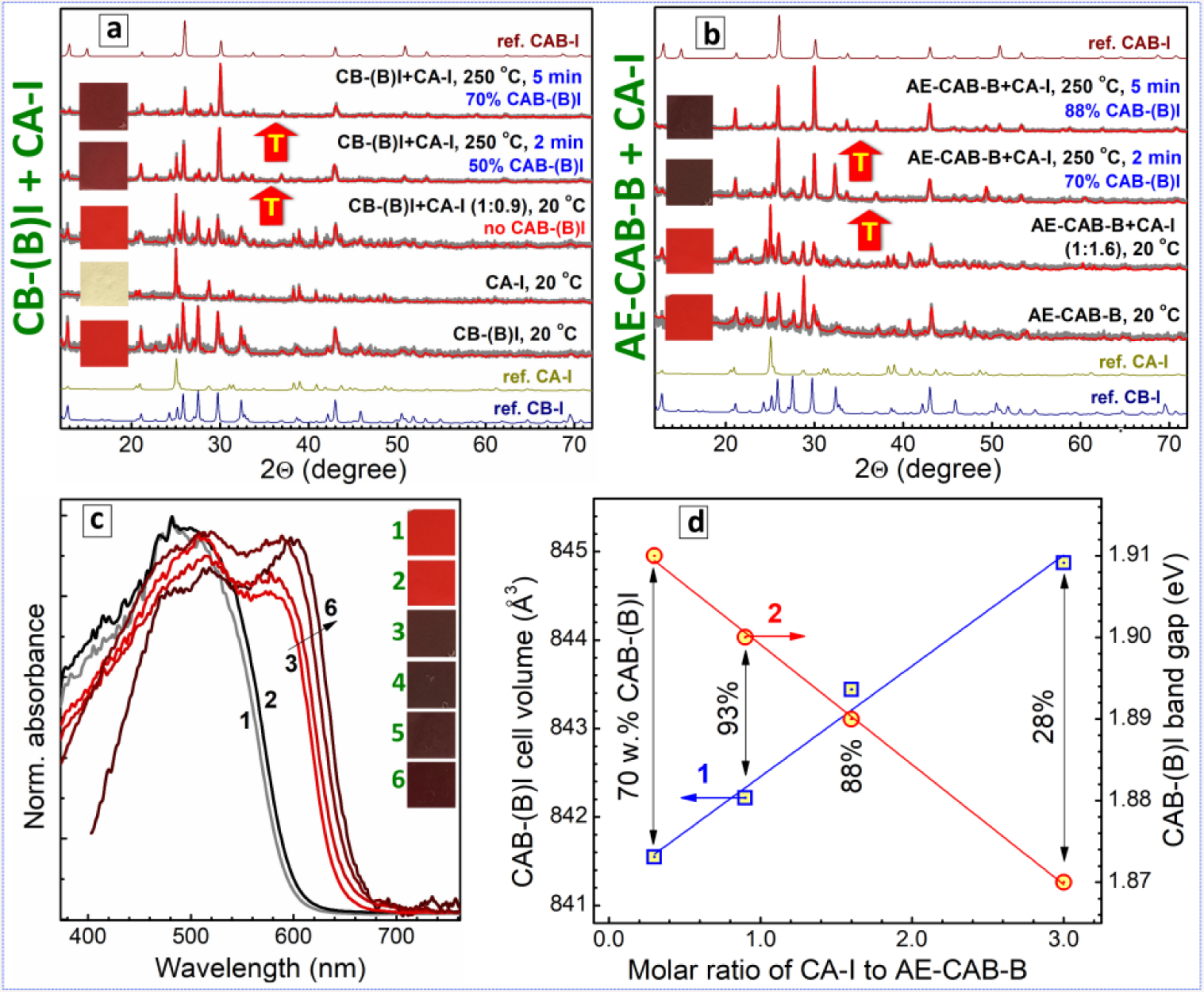
(a, b) Selected powder X-ray patterns for various systems tested in the process of CAB-(B)I perovskite formation. Gray lines correspond to experimental data; red lines correspond to Rietveld refinements. (c) Absorption spectra of AE-CAB-B (curve 1), mechanical mixture of AE-CAB-B and CA-I with molar CB-(B)­I/CA-I ratio of 1:3 (2), and products of annealing of AE-CAB-B + CA-I mixtures with molar CA-I/AE-CAB-B ratios of 0.3 (3), 0.9 (4), 1.6 (5), and 3.0 (6) at 250 °C. Inserts in (a–c) show fragments of photographs of corresponding drop-casted samples. (d) Elementary lattice cell volume (scatter 1) and band gap (scatter 2) of CAB-(B)I perovskites produced by annealing of AE-CAB-B + CA-I mixtures as functions of the molar ratio of CA-I to AE-CAB-B. The numerical values show the mass percentages of CAB-(B)I fractions in the corresponding samples. Solid lines are linear fits of the scatter data.

The crucial role of CA-I can be attributed to a lower melting point of this compound, as compared to individual CsI and AgI, which was reported to be at 210 °C.^32^ Melting of CA-I under the conditions of the present experiments is evidenced by a drastic change in the morphology upon annealing at 250 °C, with the elongated well-faceted polygonal CA-I crystals converting into much larger, molten lava-like slabs (ESI, Figure S7a). A strongly reduced intensity of PXRD reflections upon annealing attests to the amorphized glass-like state of CA-I after the annealing (ESI, Figure S7b), additionally supporting the assumption that CA-I melts during the annealing, providing a liquid reaction medium for the formation of tetragonal CAB-(B)­I. At that, CA-I retains its stoichiometry and homogeneous distribution of the constituent elements (see elemental maps in Figure S7a). Considering that both CB-(B)I and CA-I are introduced to annealing as solids, in further discussion, we will refer to the formation of CAB-(B)I as a solid-state reaction, despite the intermediate step of CA-I melting.

The solid-state reaction between CB-(B)I and CA-I can also be traced by UV–vis absorption spectroscopy (ESI, Figure S6b, curve 4). Annealing results in a “red” shift of the absorption edge of the mixture of CB-(B)I and CA-I from ca. 590 nm to ca. 630 nm, very close to the position of the absorption edge of CAB-(B)I produced by the annealing of AE-CAB-B perovskite.

### 3.2 Solid-State Reaction between AE-CAB-B and Additional CA-I

Further arguments in favor of the solid-state reaction between CB-(B)I and CA-I were collected by annealing mechanical mixtures of the as-prepared AE-CAB-B with variable amounts of CA-I. A more detailed refinement of the PXRD pattern of AE-CAB-B before annealing indicated the presence of 3–4% of CAB-(B)I in the mixture already before the thermal treatment, also indicated by a small “red” shift of the absorption spectrum of the AE-CAB-B upon mixing with CA-I (Figure 2c, curves 1, 2). Annealing of the AE-CAB-B with additional CA-I yields higher fractions of the tetragonal CAB-(B)I as compared to the CB-(B)I + CA-I mixtures, 70% versus 50% for 2 min annealing and almost 90% versus 70% for 5 min thermal treatment (Figure 2b,d).

The spectral properties of the final CAB-(B)I can be tuned by varying the molar ratio of CA-I to AE-CAB-B before annealing. In particular, the absorption band edge of CAB-(B)I perovskite shifts from ca. 640 nm to ca. 660 nm with the molar CA-I/AE-CAB-B ratio elevated from 0.3 to 3.0 (Figure 2c), corresponding to the band gap narrowing from 1.91 to 1.87 eV (Figure 2d, scatter 2). The band gap variation is most probably achieved by increasing the iodide fraction in CAB-(B)­I, evidenced by a simultaneous expansion of the elementary cell (Figure 2d, scatter 1). The increase of the CA-I/AE-CAB-B ratio from 0.3 to 1.0–1.6 results in an increase of the CAB-(B)I phase fraction from 70% up to ca. 90%, but shows in a drastic drop of the CAB-(B)I content down to ca. 30% at higher AE-CAB-B/CA-I ratios (Figure 2d). Considering the reported instability of microcrystalline CAB-I,^25,26^ this drop can be associated with a decreased stability of the CAB-(B)I phase at higher iodide fractions, settling a trade-off between the CAB-(B)I stability/content and the lowest achievable band gap. A similar stabilizing effect of residual bromide on iodide phase was recently observed also for the family of Cs_3_(Bi_*x*_Sb_1–*x*_)_2_(Br,I)_9_ double salts produced in similar conditions by the anion exchange.^28^

Summarizing this section, we show the feasibility of the thermal solid-state reaction between the products of the anion-exchange conversion of CAB-B perovskite, CB-(B)I and CA-I, resulting in tetragonal CAB-(B)­I perovskite, by synthesizing the same final product from a mechanical mixture of separately produced CB-(B)I and CA-I double salts. Considering that CB-(B)I was also synthesized by anion-exchange conversion of CB-B double salt, two alternative ways to CAB-(B)I perovskite from different bromide precursors can be realized independently, as illustrated by the scheme in Figure 3a. This scheme outlines the two-stage synthesis of CAB-(B)I involving RT AE in CAB-B or CB-B precursors, followed by thermal annealing in the presence of CA-I, the latter formed *in situ* from CAB-B perovskite (Figure 3a, case (i)) or intentionally added on the annealing stage (Figure 3a, case (ii)). We refer to this synthesis as a “basic two-stage AE/T process”, to be further expanded to more complex compositions, as discussed in Section 4.

**Figure 3:**
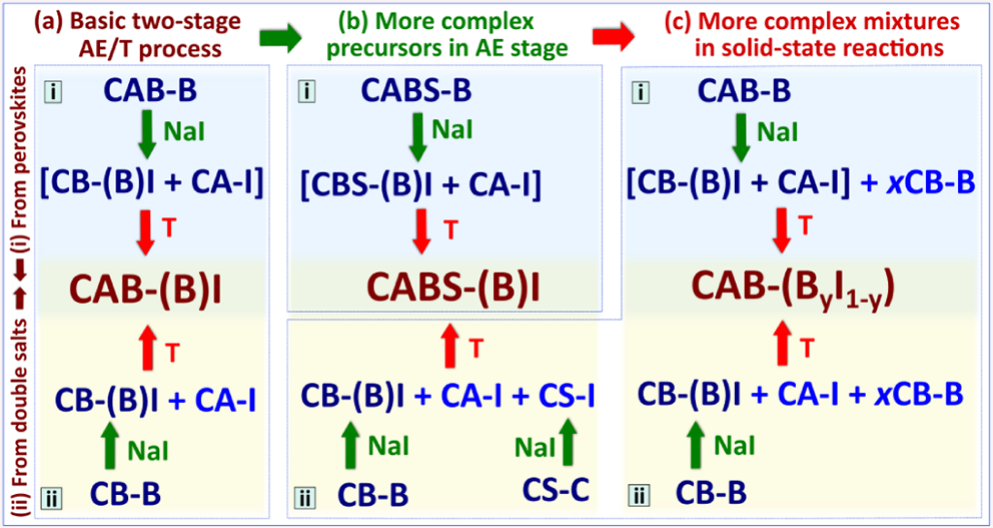
A scheme of two-stage anion-exchange/solid-state-reaction-based conversion of bromide double perovskites and double salts into tetragonal bromo-iodide perovskites. List of compositional abbreviations: CAB-B—Cs_2_AgBiBr_6_, CABS-B—Cs_2_Ag­(Bi*_x_* Sb_1–*x*_)­Br_6_, CB-B—Cs_3_Bi_2_Br_9_, CB-(B)­I—Cs_3_Bi_2_(Br,I)_9_, CA-I—CsAg_2_I_3_, CS-C and CS-I—Cs_3_Sb_2_Cl_9_ and Cs_3_Sb_2_I_9_, CAB-(B)­I and CAB-(B*_y_* I_1–*y*_)—Cs_2_AgBi­(Br,I)_6_, CABS-(B)­I—Cs_2_Ag­(Bi*_x_* Sb_1–*x*_)­(Br,I)_6_. Square brackets refer to mixtures produced by anion exchange (AE), the (B)I abbreviation indicates the presence of residual bromide (10–20%), while (B*_y_* I_1–*y*_) refers to bromo-iodide alloys with a higher and variable content of bromine.

## 4 Optimizing the Basic Two-Stage AE/T Synthesis of CAB-(B)I Perovskite

Considering the incomplete character of the solid-state conversion of AE-CAB-B into CAB-(B)I discussed in Section 3, as well as a variety of parameters that can be tuned both during the anion exchange and the subsequent annealing, a multiparametric optimization of the entire basic AE/T process was performed to find the conditions yielding the highest fraction of the CAB-(B)I phase with the lowest band gap.

### 4.1 Optimization of the Conditions of Anion Exchange

At the RT AE stage, multiple parameters can be varied, including the molar ratio of CAB-B to NaI, the water-to-2-propanol volumetric ratio in the NaI solution, and the contact time between CAB-B and the NaI solution.

The effect of the CAB-B/NaI ratio was probed by gradually increasing the amount of NaI from the nominal stoichiometric amount necessary to exchange bromide anions completely (referred to as 100%) up to almost double excess (180%). All anion-exchanged products were then annealed under identical conditions (10 min at 250 °C).

The sample produced at 100% NaI contains the highest fraction of CAB-(B)I phase, 95%, and at the same time shows the highest fraction of bromide, 40 molar%, in the halide component (ESI, Table S1), resulting in the smallest elementary cell volume and the highest band gap of 1.99 eV (Figure 4a). As the NaI amount is increased from 100% to 133%, the band gap narrows to 1.91 eV, and the actual iodide content increases to 82% of the total halide component, while the yield of the CAB-(B)I phase is still retained at a relatively high level of 65 wt.% (ESI, Table S1).

**Figure 4:**
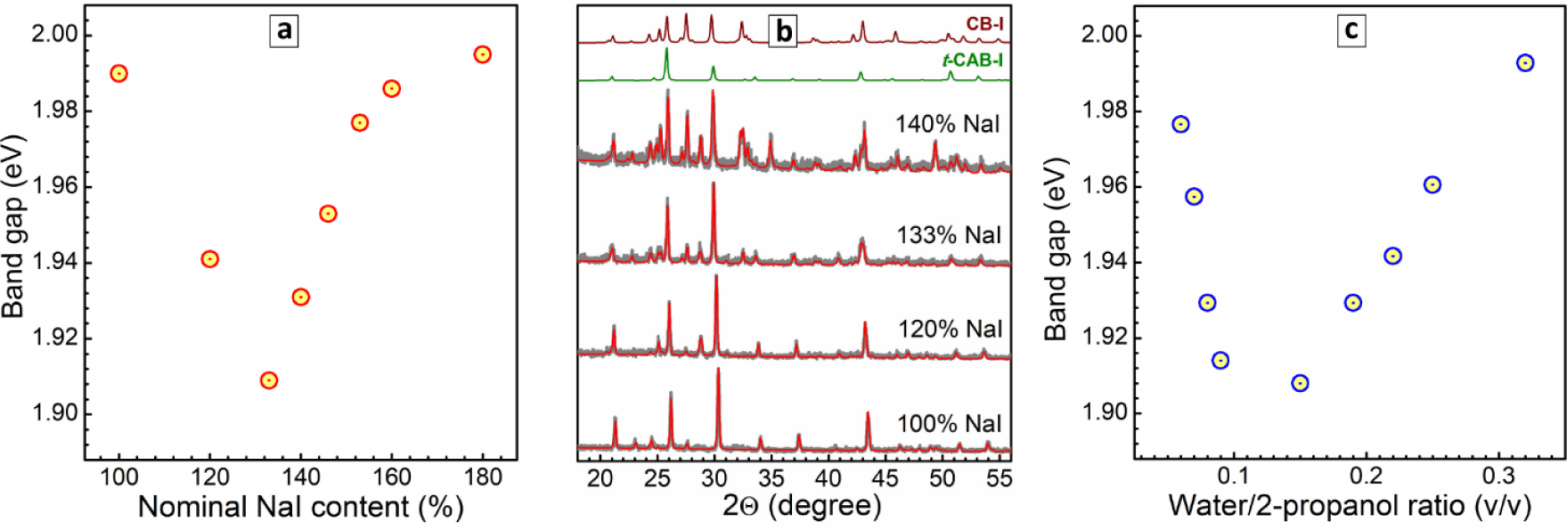
(a, c) Band gap of CAB-(B)­I samples as a function of varied NaI content (a) and varied water/2-propanol ratio at a constant NaI content of 133%. (b) Exemplary XRD profiles of annealed CAB-(B)I samples produced with 100–140% NaI content; gray lines correspond to experimental data, red lines—Rietveld refinement.

At NaI contents higher than 133%, the band gap grows again (Figure 4a), while the CAB-(B)­I fraction drops drastically to 15 wt.% for 140% NaI, further to 5 wt.% for 150% NaI, and to zero at 160% NaI (ESI, Table S1), with the hexagonal CB-(B)­I phase dominating the PXRD pattern of these products (Figure 4b). Therefore, the range of optimal content of NaI for AE is very narrow and focused at 133% NaI, when the highest mass yield and the iodide content, as well as the lowest band gap can be simultaneously achieved for the tetragonal CAB-(B)­I.

In the currently adopted synthetic protocol, increasing NaI content is accompanied by an increase in the amount of water added to the system, necessitating separate analyses of the effects of NaI and water. The composition of the solvent for NaI solution was optimized to achieve the highest solubility of NaI, as well as NaBr as the AE product to be removed, at the same time minimizing the solubility of other compounds, including CAB-B, CB-(B)­I, and CA-I. In variations of NaI content, an excess of water introduced with NaI is expected to induce hydrolytic processes on the surface of the CB-(B)I crystals, passivating the surface and preventing reaction with CA-I upon annealing.

This possibility was tested on a series of NaI solutions with a constant relative sodium iodide content of 133% and a varying water-to-2-propanol volumetric ratio. It was found that increasing the water/2-propanol ratio above 0.15 leads to a rapid increase in the band gap of the final products (Figure 4c) and a reduction in the CAB-(B)I fraction in the products. These observations indicate that the subsequent thermal solid-state reaction between CB-(B)I and CA-I is hindered, most likely due to hydrolytic passivation of the CB-(B)I surface. An increase in the band gap was also observed at water/2-propanol ratios smaller than 0.1, most probably due to a low solubility of NaBr (as an AE byproduct) and a low AE efficiency. A similar increase in the band gap of the final CAB-BI products was also observed for longer periods of the contact between the CAB-B crystals and NaI solution, with the NaI content and water/2-propanol ratio kept constant (ESI, Figure S8). This observation also supports the possibility that hydrolytic processes occur concurrently with AE and become more likely as contact time increases.

### 4.2 Optimization of the Conditions of Thermal Solid-State Reaction

The outcomes of the thermal treatment of the [CB-(B)I + CA-I] mixtures can be strongly affected by variations of the annealing duration at a constant T, or, *vice versa*, by variations of the annealing temperature at a constant duration.

The annealing duration of the AE-CAB-B produced at 133% NaI was varied at a constant annealing temperature (250 °C) from 1 to 20 min. As discussed above, a few percent of the CAB-(B)I phase can be found even before the annealing, with the CAB-(B)I fraction rapidly growing up to ca. 40% even after the first minute of the thermal treatment and further to ca. 60% for 10 min annealing (Figure 5a,b).

**Figure 5:**
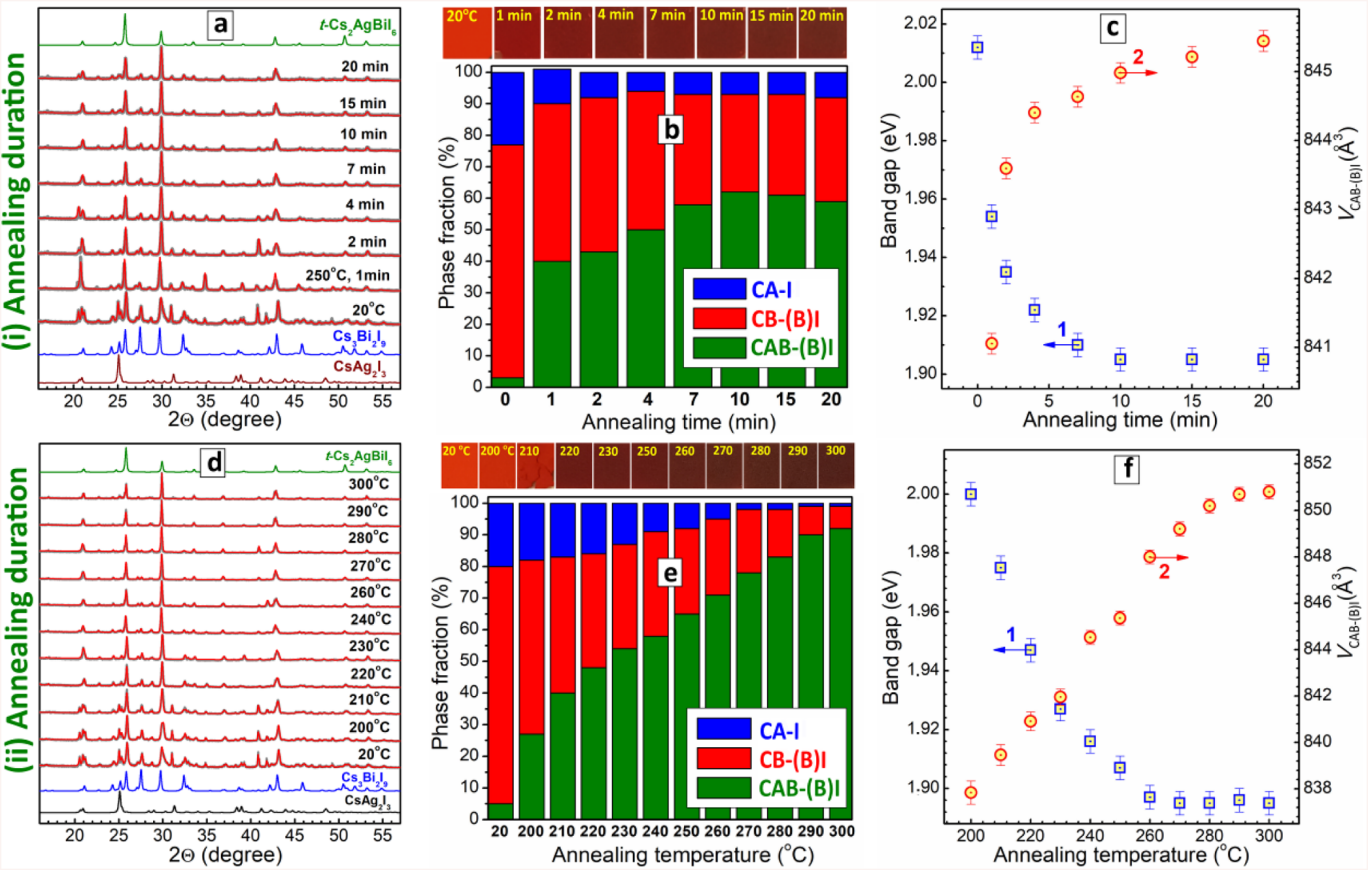
(a, d) Powder XRD profiles and (b, e) phase distributions for AE-CAB-B at RT and after annealing at 250 °C for for 1–20 min (a, b), or for 10 min at 200–300 °C (d, e). Gray and red lines in (a, d) show experimental data and Rietveld refinement, respectively. Photographs in the upper part of (b, e) show the evolution of the sample color. (c, f) Evolution of the band gap (scatter 1) and elementary cell volume (scatter 2) of CAB-(B)I with the annealing time (c) and temperature (f).

The increase of the CAB-(B)I phase content is accompanied by a narrowing of the band gap (Figure 5c, scatter 1) and an expansion of the elementary cell (Figure 5c, scatter 2), indicative of an increasing iodide fraction in the halide sublattice. Longer annealing times, 15–20 min, do not contribute noticeably to the yield of the CAB-(B)I phase (Figure 5b) and do not further affect the band gap (Figure 5c, scatter 1), while providing only a marginal increment of *V*_CAB‑(B)I_.

The temperature of annealing was found to be a vital factor affecting the yield of the CAB-(B)I phase, which grows from ca. 25% at 200 °C up to 90–92% at 290–300 °C (Figure 5d,e). A further increase in temperature was not pursued because the drop-cast CAB-BI samples detached extensively from the glass substrate at higher temperatures. Still, the probed temperature range attests to the remarkable thermal stability of CAB-(B)­I perovskites and to their resistance to oxidation by ambient oxygen.

Both the elementary cell volume and the band gap of CAB-(B)I perovskites continuously evolve at the elevation of the annealing temperature from 200 °C up to ca. 280 °C, and saturate at higher temperatures (Figure 5f). At that, the band gap narrows from 2.0 eV for the sample prepared at 200 °C to slightly below 1.9 eV for CAB-(B)I compounds produced at 260 °C, showing no further decrease at higher temperatures (Figure 5f, scatter 1).

The elementary cell volume reveals a steady growth from 838 Å^3^ at 200 °C to 848 Å^3^ at 260 °C, followed by a slow growth until 851 Å^3^ at 300 °C (Figure 5f, scatter 2). The cell expansion indicates a gradual increase of the iodide fraction in the halide component of the CAB-(B)I perovskite (see ESI, Table S1). In particular, the product collected after the annealing at 300 °C revealed the stoichiometry of double perovskite and a bromide fraction of 12% (Table 1, sample ID “CAB-(B)­I, 300 °C”). We note that this series of samples was selected as an example to track the evolution of the quality and goodness parameters of Rietveld refinement for multiphase mixtures, as shown in the ESI (Figure S9 and Table S2).

In summary, the optimizations of both steps of the synthesis of CAB-(B)I perovskites—the room-temperature anion-exchange and the subsequent annealing—identified a set of synthesis parameters resulting in the highest yield of the tetragonal CAB-(B)I perovskite, ca. 90%, with the highest iodide content of ca. 90% and the lowest indirect band gap of ca. 1.9 eV. Together with the optimized RT AE conditions, these parameters were incorporated into the basic AE/T process illustrated in Figure 3a.

### 4.3 The Synthesis of Phase-Pure CAB-(B)I Is Feasible

As shown in Sections 4.2 and 4.3, the yield of CAB-(B)I perovskites remains below 100% even in the fully optimized conditions, with the final products containing ca. 10 mass% of unreacted CB-(B)I and CA-I double salts. The incomplete character of the solid-state reaction could be a natural limitation of the proposed AE/T approach due to the reverse process (1), but could also reflect kinetically limited transformation of nonideally mixed precursors and limited accessibility of CA-I, present as larger well-separated crystals (see Figure 1d and SI, Figure S7).

To distinguish between these two limitations and probe the feasibility of the synthesis of phase-pure CAB-(B)­I products, we modified the basic (fully optimized) AE/T process by adding steps of mechanical grinding before and after the thermal solid-state reaction, as well as an additional annealing after the second grinding (Figure 6, right panel).

**Figure 6:**
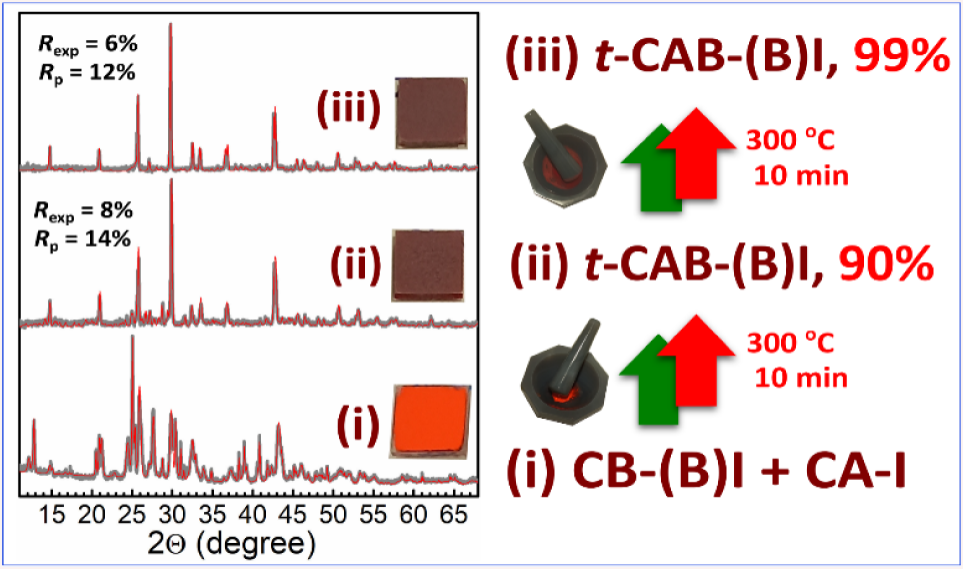
Powder XRD patterns of a starting mixture of CB-(B)I and CA-I double salts after mechanical grinding (i), tetragonal Cs_2_AgBi­(Br,I)_6_ perovskite (*t*-CAB-(B)­I) formed after the first annealing of such mixture at 300 °C for 10 min (ii), and *t*-CAB-(B)I produced by an additional step of mechanical grinding of (ii) and the second annealing at 300 °C for 10 min (iii). The gray and red lines show experimental data and Rietveld refinements, respectively. The schematic on the right panel illustrates the two-annealing pathway to the phase-pure *t*-CAB-(B)­I.

The annealing of a mechanically ground mixture of CB-(B)I and CA-I (Figure 6, pattern (i)) at 300 °C yields tetragonal *t*-CAB-(B)­I perovskite with a yield of 90% (Figure 6, pattern (ii)), that is, almost the same, as in the case of the solid-state reaction without the grinding of the precursors (87%, see ESI, Figure S10, patterns (i, (ii))).

However, the second mechanical grinding of the *t*-CAB-(B)I products, followed by an additional annealing at 300 °C was found to eliminate the under-reacted double salts almost completely and yield phase-pure *t*-CAB-(B)I with the same lattice parameters and only scarcely detectable residuals of the double salt precursors of less than 1% (Figure 6, pattern (iii)). At that, the second annealing, performed without the intermediate mechanical grinding step, shows a lower *t*-CAB-(B)I yield of only 95%. The details of the two-stage annealing/grinding are provided in ESI (Materials and Methods, section III).

In summary, the above-discussed experiments show the practical feasibility of producing phase-pure tetragonal CAB-(B)I perovskites by an additional postsynthesis grinding/annealing step, as illustrated by the scheme in Figure 6 (right panel), indicating that the impurities in the final *t*-CAB-(B)I originate from kinetic limitations of the solid-state reaction, rather than from the inherent decomposition of *t*-CAB-(B)I into original precursors, similar to reaction (1).

Remarkably, the substitution of relatively gentle manual grinding in an agate mortar by a more intense mechanochemical treatment in a planetary ball mill produced an adverse effect on the properties of the final products. The products of the ball-milling were found to show reduced reflections of *t*-CAB-(B)I and appearance of additional reflections (ESI, Figure S10, pattern (iii)), indicating a partial decomposition of the perovskite. The annealing of the ball-milled products results in complex mixtures of multiple phases (ESI, Figure S10, pattern (iv)), rather than the target phase-pure product, most likely due to partial oxidation and hydrolysis of *t*-CAB-(B)­I during the mechanical treatment.

## 5 From Basic AE/T Process to a Broader Scope and a Higher Complexity of Precursors

The above-discussed basic RT AE/T process of converting different bromide precursors into CAB-(B)I perovskites was so far limited to bismuth-based precursor compounds and provided only a marginal control over the composition of the halide sublattice. As the next step, we explored the applicability of this approach to more complex precursor combinations, yielding more complex and better-controlled perovskite products, and established a general synthetic pathway for bromoiodide double perovskites with variable cationic-metal and anionic-halide sublattices.

In the two-stage synthesis, greater product complexity can be achieved either by introducing more complex perovskite precursors to anion-exchange-based transformations or by subjecting more complex combinations of precursor double salts to thermal annealing, converting them into tetragonal double perovskites. The first pathway can be integrated with the second one by employing anion exchange to tailor the synthesis of more complex double-salt precursors, which are then subjected to solid-state reactions with other components.

### 5.1 More Complex Precursors for AE: The Case of Cs_2_Ag­(Bi_*x*_Sb_1–*x*_)­Br_6_

Recently, we reported a band-bowing effect observed for many halide compounds with a mixture of Bi^3+^ and Sb^3+^ on the M^3+^ site, including Cs_2_Ag­(Bi_*x*_Sb_1–*x*_)­(Cl,Br)_6_ perovskites^27,30^ and Cs_3_(Bi_*x*_Sb_1–*x*_)_2_X_9_ double salts, X = Cl, Br, and I.^28^ The band gaps of Bi/Sb-alloyed halides were found to be lower than the band gaps of the corresponding Bi-pure and Sb-pure halides, with the minimal *E*_g_ values observed close to *x* = 0.5 and the bowing parameter depending on the composition of the halide subsystem.^28^ The above-discussed completely optimized basic AE/T process yields CAB-(B)I perovskites with the lowest bandgap limited to ca. 1.9 eV, with the stability of CAB-(B)I perovskite drastically deteriorating at higher iodide contents, which potentially can provide lower band gaps. In this situation, further narrowing of the band gap may be possible through a broader compositional design, for example, by combining Bi^3+^ and Sb^3+^ at the crystallographic M^3+^ site of the tetragonal double perovskite. To this aim, we explored the feasibility of applying the two-stage AE/T process to more complex Cs_2_Ag­(Bi_*x*_Sb_1–*x*_)­Br_6_ precursors (CABS-B).

#### 5.1.1 Synthesis of CABS-B

The earlier-developed RT synthesis protocol for the CAB-B perovskite was extended with minimal modifications to combinations of Bi^3+^ and Sb^3+^ (see details in the ESI), yielding a series of CABS-B products with varying Bi/Sb ratios as microcrystalline powders (ESI, Figure S11). Similar to previous studies on mixed Bi/Sb halides,^27,28,30^ the CABS-B series exhibited visual evidence of band bowing (see photographs in Figure 7a and Figure S11). However, an analysis of the PXRD profiles of the series showed all Bi/Sb-mixed samples to be multiphase products, containing CAB-SB perovskite, trigonal Cs_3_(Bi_*x*_Sb_1–*x*_)_2_Br_9_ double salt (CBS-B), and AgBr in different proportions (Figure 7a). The content of double CABS-B perovskite was found to gradually decrease from 98 wt.% for 20% Sb to below 20 wt.% for 100% Sb (Figure 7b). At that, both CABS-B perovskite and CBS-B double salt followed linear dependences of the lattice parameter (elementary cell volume) on the nominal Sb fraction (Figure 7c), indicating both products to be Bi/Sb-mixed solid solutions. In line with XRD observations, the SEM inspection of CABS-B samples with *x* < 0.6 (ESI, Figure S11) reveals the presence of larger polygonal crystals and microrods, which can be tentatively assigned to AgBr and CBS-B, respectively.

**Figure 7:**
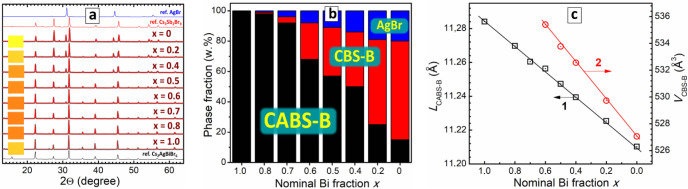
(a) Powder XRD patterns, (b) phase distribution, and (c) lattice parameters of CABS-B (scatter 1) and CBS-B (scatter 2) in Cs_2_Ag­(Bi*_x_* Sb_1–*x*_)­Br_6_ products with a variable Bi fraction. In (a), inserts show photographs of corresponding samples as drop-cast films on glass; gray and red lines represent experimental data and Rietveld refinements, respectively. In (c), solid lines represent linear fits of experimental data.

All CABS-B products showed a good correspondence between the nominal Sb fraction (set at the synthesis) and the actual Sb fraction in final products, as exemplified in Table 1 for Cs_2_AgBi_0.5_Sb_0.5_Br_6_. Though being a multiphase mixture, this product showed a formal stoichiometry of a double perovskite with Br/M, Cs/M, and Ag/M ratios close to 6, 2, and 1, respectively (M = Bi + Sb, Table 1, sample ID “CABS-B, nom. Bi:Sb = 1:1”).

#### 5.1.2 Anion-Exchange Transformations of CABS-B

Similar to the above-discussed case of CAB-B, the anion exchange with NaI (133%) yields microcrystalline mixtures with two distinct crystal morphologies, exemplified in Figure 7a for Bi:Sb = 1:1. A site-selective EDX analysis showed the fraction of smaller microcrystals with a grain size below 1 μm (site 1 in Figure 8a) to be Cs_3_(Bi_0.5_Sb_0.5_)_2_(Br,I)_9_ double salt (CBS-(B)­I) containing 22% of residual bromide (Table 1, sample ID “AE-CABS-B”). Larger microrod-shaped crystals (site 2 in Figure 8a) were identified by EDX as CsAg_2_I_3_ (Table 1), consistent with the elemental EDX mapping of AE-CABS-B, which shows microrods enriched in silver (Figure 8a).

**Figure 8:**
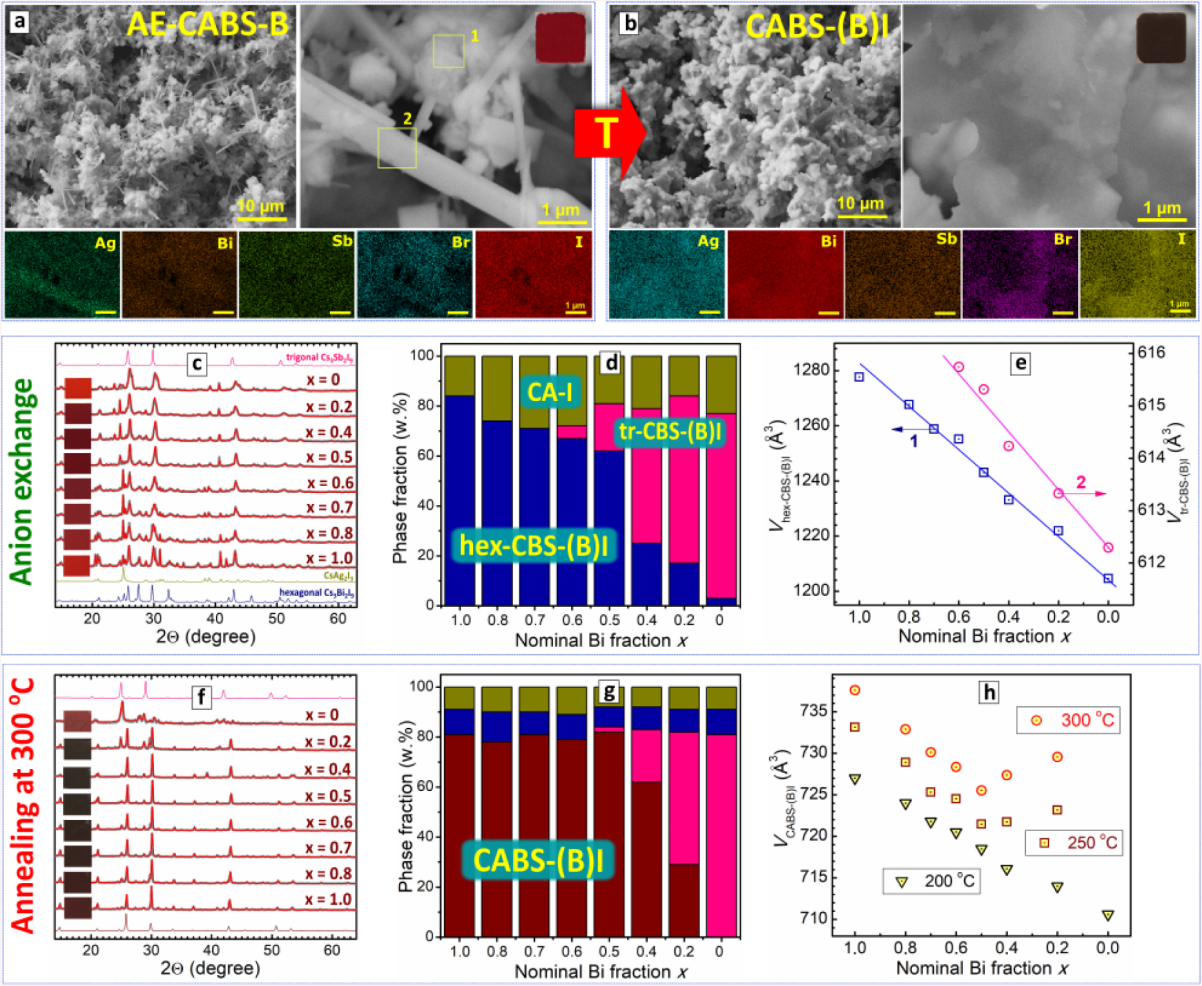
(a, b) SEM images and element distribution maps for Ag, Bi, Sb, Br, and I for the products of anion exchange of CABS-B (AE-CABS-B) with NaI at 20 °C (d) and their thermal conversion into CABS-(B)­I by the open-air annealing at 300 °C (e). In (a), rectangles (1) and (2) mark two distinct morphologies selected for EDX analysis (see Table 1). (c–h) Powder XRD patterns (c, f), phase distributions (d, g), and lattice parameters of hexagonal CBS-(B)I (scatter 1) and trigonal CBS-(B)I (scatter 2) components of AE-CABS-B (e), and CABS-(B)I produced at different temperatures (h). In (c, f), inserts show photographs of corresponding samples as drop-cast films on glass; gray and red lines represent experimental data and Rietveld refinements, respectively. In (e), solid lines represent linear fits of experimental data.

The EDX assessments made for a selected AE-CABS-B sample with *x* = 0.5 are supported by a PXRD overview of the entire series of anion-exchanged products with variable Bi fraction (Figure 8c). The best fits of the PXRD profiles were obtained by assuming that the samples are mixtures of hexagonal and trigonal CBS-(B)I double salts, and CA-I (Figure 8d). Both hexagonal and trigonal CBS-(B)I compounds showed linear dependencies of the elementary cell volume on the nominal Sb fraction (Figure 8e), indicating the formation of ideal solid solutions. The distributions of hexagonal and trigonal CBS-(B)I double salt for different Bi fractions (Figure 8d) mirror, to some extent, the distribution of the CABS-B and CBS-B phases in the bromide precursors (Figure 7b). This similarity indicates that the hexagonal and trigonal CBS-(B)I phases originate from the anion exchange in CABS-B and CBS-B phases, respectively.

#### 5.1.3 Annealing-Induced Transformations of AE-CABS-B

To simultaneously assess the effect of Bi fraction and annealing temperature on the structural and spectral properties of CABS-(B)­I products, the AE-CABS-B series with variable Bi fraction was replicated four times and subjected to annealing at 150 °C, 200 °C, 250 °C, and 300 °C (Figure 9a, series “+ thermal annealing”).

**Figure 9:**
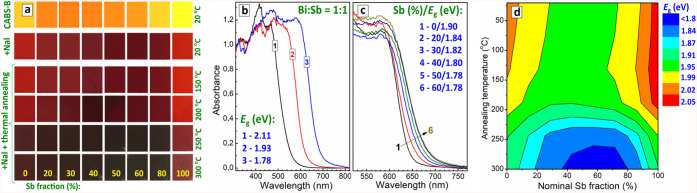
(a) A set of photographs (fragments) of CABS-B samples, products of their anion exchange with NaI and annealing of anion-exchanged samples at different temperatures. (b) Exemplary absorption spectra of original CABS-B produced at *x* = 0.5 (curve 1), and AE-CABS-B before (2) and after annealing at 300 °C (3). (c) Evolution of the absorption spectrum of CABS-(B)I with increasing Sb fraction. (d) Band gap mapping of CABS-(B)I produced at different Sb fractions and annealing temperatures.

The PXRD patterns of the AE-CABS-B series annealed at 300 °C (Figure 8f) reveal the dominance of the tetragonal Cs_2_Ag­(Bi_*x*_Sb_1–*x*_)­(Br,I)_6_ perovskite phase (further denoted as CABS-(B)­I), ca. 80 wt.%, in the samples with the Sb content up to 50% (Figure 8g). The rest of the sample, ca. 20 wt.%, was identified as a mixture of AgI, CA-I, and trigonal CBS-(B)­I.

A more detailed SEM/EDX characterization was performed for the exemplary CAB-(B)I sample produced at nominal *x* = 0.5 and 300 °C. Similar to the above-discussed CAB-(B)I case, the annealing of AE-CABS-B yields molten lava-like densely packed products with no distinct grain sizes (Figure 8b). This product shows almost perfect stoichiometry of double perovskite, and the actual Bi fraction, 0.49, closely matches the nominal one (Table 1, sample ID “CABS-(B)­I, 300 °C”). The elemental mapping shows uniform distributions of Bi, Sb, Br, and I (Figure 8b), indicating homogeneous alloying of all elements, consistent with PXRD data.

At Bi fractions lower than 50%, the content of the tetragonal CABS-(B)­I phase drops drastically, from 60 wt.% for *x* = 0.4 to 30 wt.% for *x* = 0.2 and finally to zero for the Sb-pure sample (Figure 8g). Again, the distributions of the trigonal CBS-(B)I phases as functions of *x* are quite similar for the AE-CABS-B and annealed CABS-(B)I samples, indicating that the trigonal CBS-(B)I does not participate in the solid-state reaction with CA-I.

Alternatively, the presence of the trigonal CBS-(B)I phase in Sb-rich samples can be explained by the partial decomposition of the CABS-(B)­I perovskite upon cooling after thermal treatment. In favor of such an interpretation is the nonmonotonic variation in the elementary cell volume of CABS-(B)I perovskites produced at 300 °C with *x* (Figure 8h, circles). The *V*_CABS‑(B)I_ decreases linearly with increasing Sb fraction up to 50%, due to the smaller ionic radius of Sb^3+^ relative to Bi^3+^, consistent with the behavior expected for ideal solid solutions. Then, at higher Sb content, *V*_CABS‑(B)I_ begins to grow, coinciding with the appearance of the trigonal CBS-(B)I phase in the samples (compare Figure 8g and h). These trends can be interpreted as the results of a partial exclusion of Sb^3+^ from the CABS-(B)I perovskite into a separate CBS-(B)I phase, resulting in an enrichment of the tetragonal CABS-(B)I perovskite with Bi^3+^ and the growth of *V*_CABS‑(B)I_. The same trends are observed for the samples annealed at 250 °C (Figure 8h, squares). However, for the samples produced at 200 °C, a linear dependence of *V*_CABS‑BI_ on the Sb content is retained for the entire range of antimony fractions (Figure 8h, triangles), though the content of the CABS-(B)­I phase remains low in such conditions.

In general, the *V*_CABS‑(B)I_ systematically increases with annealing temperature for each sample, indicating a higher iodide content in the CABS-BI perovskites produced at higher temperatures. Indeed, the exemplary CABS-(B)I perovskite with *x* = 0.5 showed a bromide residual of 12% (Table 1), while similar perovskites produced at 250 °C typically show the presence of 20–22% of bromide.

The conversion of AE-CABS-B into tetragonal CABS-(B)­I is accompanied by a distinct change of the sample color (compare inserts in Figure 8a and b) and a considerable “red” shift of the absorption band edge. In the exemplary case of CABS-(B)I with *x* = 0.5, the RT AE-driven conversion of CABS-B precursor shifts the absorption threshold from ca. 550 nm to ca. 620 nm (Figure 9b, curves 1, 2), with a further “red” shift to ca. 680 nm achieved after the annealing of AE-CABS-B at 300 °C (Figure 9b, curve 3).

Similar to CAB-B and CAB-(B)­I, the absorption spectra of CABS-B and CABS-(B)I perovskites demonstrate extended linear sections when presented in Tauc coordinates for both indirect and direct allowed electronic transitions (ESI, Figure S12). In terms of indirect transitions, as a more general case, the evolution of the absorption spectrum presented in Figure 9b (curves 1, 3) corresponds to a narrowing of the band gap from 2.11 to 1.78 eV.

Figure 8c summarizes the evolution of the band gap of CABS-(B)I perovskites as a function of antimony content and annealing temperature. The *E*_g_ mapping indicates that Bi/Sb alloying shifts the absorption band to lower energies by 120 meV, thereby increasing the potential of such perovskites as solar absorbers, particularly for tandem or indoor PV.^11^ The map reveals a “valley” of the band gaps going below 1.8 eV, for the samples with 40–80% Sb annealed at ca. 270–300 °C (Figure 9d).

### 5.2 More Complex Mixtures for Annealing: The Cases of Cs_2_Ag­(Bi_*x*_Sb_1–*x*_)­(Br,I)_6_ and Cs_2_AgBi­(Br_*y*_I_1–*y*_)

The feasibility of forming tetragonal CAB-(B)I perovskites by annealing mixtures of individual double salts, CB-(B)I with CA-I, as illustrated in case (ii) in Figure 3a, or mixtures of anion-exchanged bromide perovskites with CA-I, indicates that a general combinatorial approach to perovskites with tailored composition as well as with a higher compositional complexity can be achieved by introducing more complex and versatile mixtures of double salt precursors into the thermally activated solid state reactions with CA-I. In the present section, we explore this venue on two case examples: by combining Bi^3+^ and Sb^3+^ in tetragonal Cs_2_Ag­(Bi_*x*_Sb_1–*x*_)­(Br,I)_6_ perovskites and by alloying bromide and iodide anions in a series of tetragonal Cs_2_AgBi­(Br_*y*_I_1–*y*_)_6_ perovskites, both produts produced from mechanical mixtures of simpler halide double salts.

#### 5.2.1 Producing CABS-(B)I from Double Salts

The above-discussed two-stage synthesis of tetragonal CABS-(B)­I perovskites, illustrated by case (i) in Figure 3b, is very similar to the basic AE/T process developed for a simpler CAB-B precursor (case (i) in Figure 3a). In this context, it can be expected that the alternative pathway to CAB-(B)­I, starting from individual double CB-(B)I and CA-I salts (case (ii) in Figure 3a), would also be possible for antimony-containing double salt precursors.

The feasibility of such reactions was tested by annealing mechanical mixtures of CB-(B)­I with CA-I and Cs_3_Sb_2_I_9_ (CS-I), the latter of which was produced from Cs_3_Sb_2_Cl_9_ (CS-C) via anion exchange with NaI.^28^ At that, the AE-conversion of CS-C yields pure hexagonal CS-I,^28^ avoiding the above-discussed bottleneck of the low reactivity of trigonal CS-(B)­I, produced by AE from CS-B, with CA-I.

The annealing of equimolar mixtures of CB-(B)I and CS-I with an excess of CA-I (Figure 10a) at 300 °C indeed results in the formation of tetragonal CABS-(B)I perovskite with a mass yield of 85% (ESI, Table S3). The perovskite reveals a characteristic molten morphology with under-resolved individual crystallites and a homogeneous distribution of Bi and Sb, as well as Br and I (Figure 10b). The CABS-(B)I perovskite shows an elementary lattice cell volume of 845 Å (Figure 10c), which is close to the cell volume of bismuth-only CAB-(B)­I, most probably due to a compensation of smaller ionic radius of Sb^3+^, by a somewhat larger content of iodide in CABS-(B)­I, 92%, as compared to CAB-(B)I (83%, see Table S3). The actual fraction of antimony in CABS-(B)­I, 56% (Table S3), is close to the one expected for the equimolar mixture of CB-(B)I and CS-I precursors. The overall process is schematically shown in case (ii) of Figure 3b.

**Figure 10:**
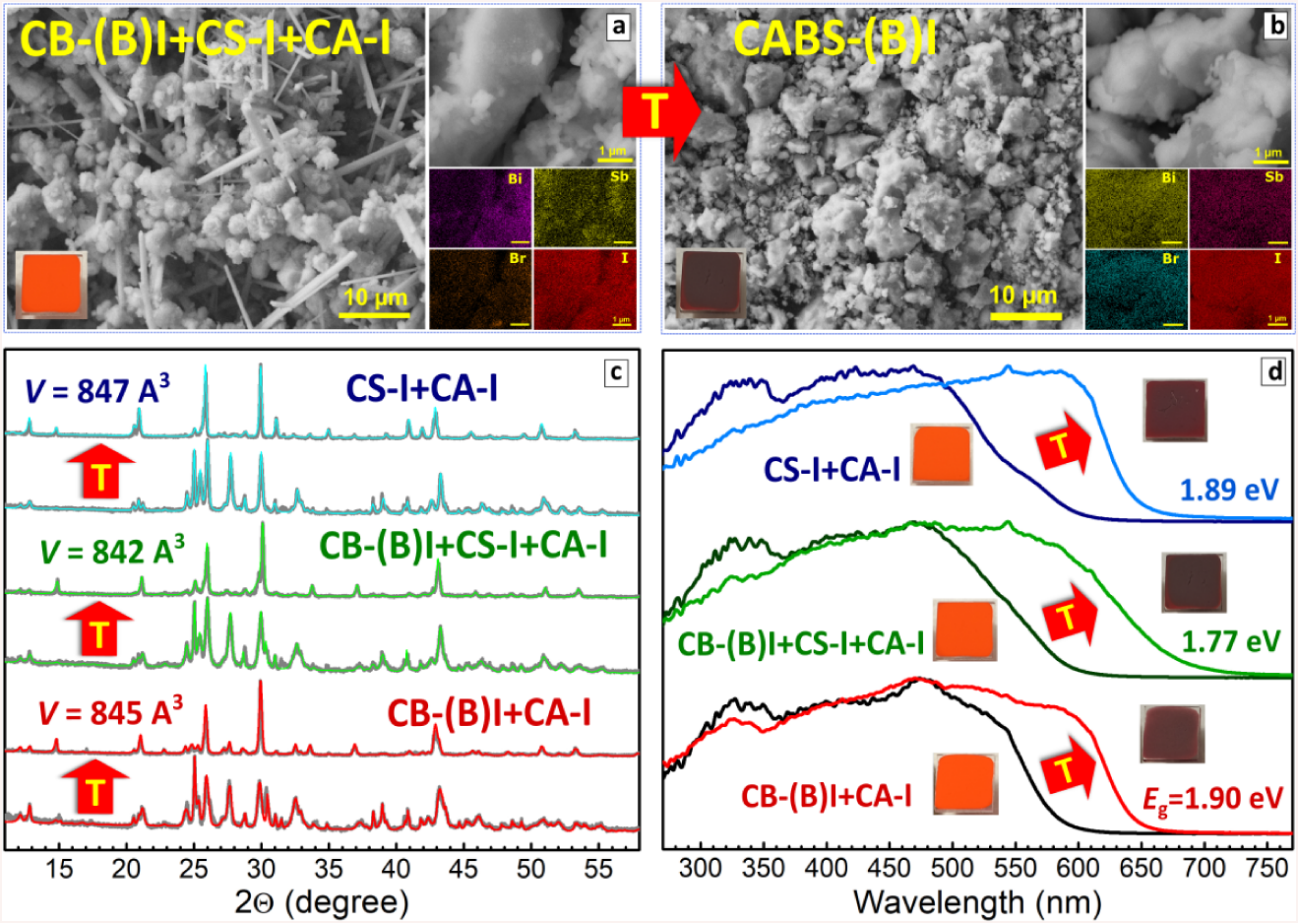
(a, b) Evolution of morphology and element distribution upon thermal conversion of a CB-(B)I + CS-I + CA-I mixture (a) into tetragonal CABS-(B)I perovskite. (c, d) Powder XRD profiles (c) and absorption spectra (d) of CB-(B)I + CA-I, CS-I + CA-I, and CB-(B)I + CS-I + CA-I mixtures and the products of their thermal conversion at 300 °C. In (c), gray lines correspond to experimental XRD data, red/green/blue lines—to corresponding Rietveld refinements; in (d), photographs show starting mixtures of double salts and final perovskites as drop-cast films.

The double CS-I salt alone also revealed a similar reactivity toward CA-I, converting upon annealing into an antimony-pure tetragonal Cs_2_AgSbI_6_ (CAS-I) perovskite (Figure 10c) with a cell volume of 847 Å with a mass yield of 95% (ESI, Table S3). The similar cell volumes of CABS-(B)I and CAS-I perovskites can also be accounted for by a compensation of the smaller cationic radius of Sb^3+^ by the larger fraction of iodide in CAS-I, as compared to CABS-(B)­I. It can be hypothesized that this compensation effect allows the tetragonal lattice motif to be preserved from CAB-(B)I to CABS-(B)I to CAS-I compounds. We note that the first report on the experimental synthesis of CAS-I perovskite as colloidal ligand-capped NCs was published in 2025 by D. Gamelin’s group, by using a similar strategy of anion-exchange-driven conversion of a bromide double perovskite precursor.^26^ At the same time, the ambiguity between the cubic and tetragonal structures of CAS-I NCs was not decisively resolved in ref 26, as earlier for the case of CAB-I NCs.^25^ While the definitive identification of the lattice type of CAS-I would require further in-depth characterizations, in particular, by using synchrotron excitation for the powder diffraction studies,^25^ the high similarity of the powder XRD patterns of CAB-(B)­I, CABS-(B)­I, and CAS-I observed in the present work (Figure 10c) allows the assignment of CAS-I to the family of tetragonal perovskites as a viable preliminary evaluation.

In all three cases, the solid-state reaction between the precursors, initiated by annealing, results in significant spectral changes, particularly “red” shifts of the absorption band edges, observable as a color change from bright orange to deeply dark brown (Figure 10d). The CAB-(B)­I and CAS-I perovskites showed very close indirect band gaps, 1.90 and 1.89 eV, respectively, while the mixed CABS-B­(I) perovskite revealed an expected band-bowing behavior absorbing in a longer-wavelength range down to ca. 700 nm, with an indirect band gap of 1.77 eV (Figure 10d; ESI, Table S3), similar to the above-discussed CABS-(B)I perovskites produced by the two-stage process from CABS-B. We note that the band gap observed for CAS-I in the present work is slightly higher than that earlier evaluated for CAS-I NCs (ca. 1.8 eV),^26^ most probably due to a stabilizing effect of surface ligands on colloidal perovskite NCs.

#### 5.2.2 Synthesis of Cs_2_AgBi­(Br_*y*_I_1–*y*_) Perovskites from Ternary Mixtures of Double Salts

Another illustration of a general character of the reported solid-state reaction between double halide salts was provided by thermally activated alloying of ternary mixtures of CB-(B)­I, CB-B, and CA-I precursors, resulting in a series of solid-solution tetragonal bromo-iodide CAB-(Br_*y*_I_1–*y*_) perovskites with largely variable bromide fraction y, as schematically depicted by case (ii) in Figure 3c. Similar effects of bromide enrichment and conversion of CAB-(B)I into CAB-(B_*y*_I_1–*y*_) can also be achieved by annealing mixtures of as-prepared AE-CAB-B with CB-B, case (i) in Figure 3c (the structural and spectral data available, but not presented to avoid confusion).

The solid-state reactions between CB-(B)­I, CB-B, and CA-I were tested for a series of mechanical mixtures with a CB-(B)­I/CB-B ratio varied from 0:5 to 5:0, and the amount of CA-I was stoichiometric to the total amount of bismuth-based double salts. The mixtures were transformed into suspensions in 2-propanol, drop-cast on glass substrates, and subjected to open-environment annealing at 300 °C (Figure 11a). The exemplary Rietveld refinement of PXRD in Figure 11b shows that the annealing results in a solid-state conversion of the mixture of double salts (pattern 1) into a tetragonal CAB-(B_*y*_I_1–*y*_) perovskite (pattern 2) with a cell volume of 802 Å and a mass yield of 92% (see the data for the whole series in ESI, Table S4; collections of SEM images and exemplary EDX spectra in Figures S13 and S14, respectively).

**Figure 11:**
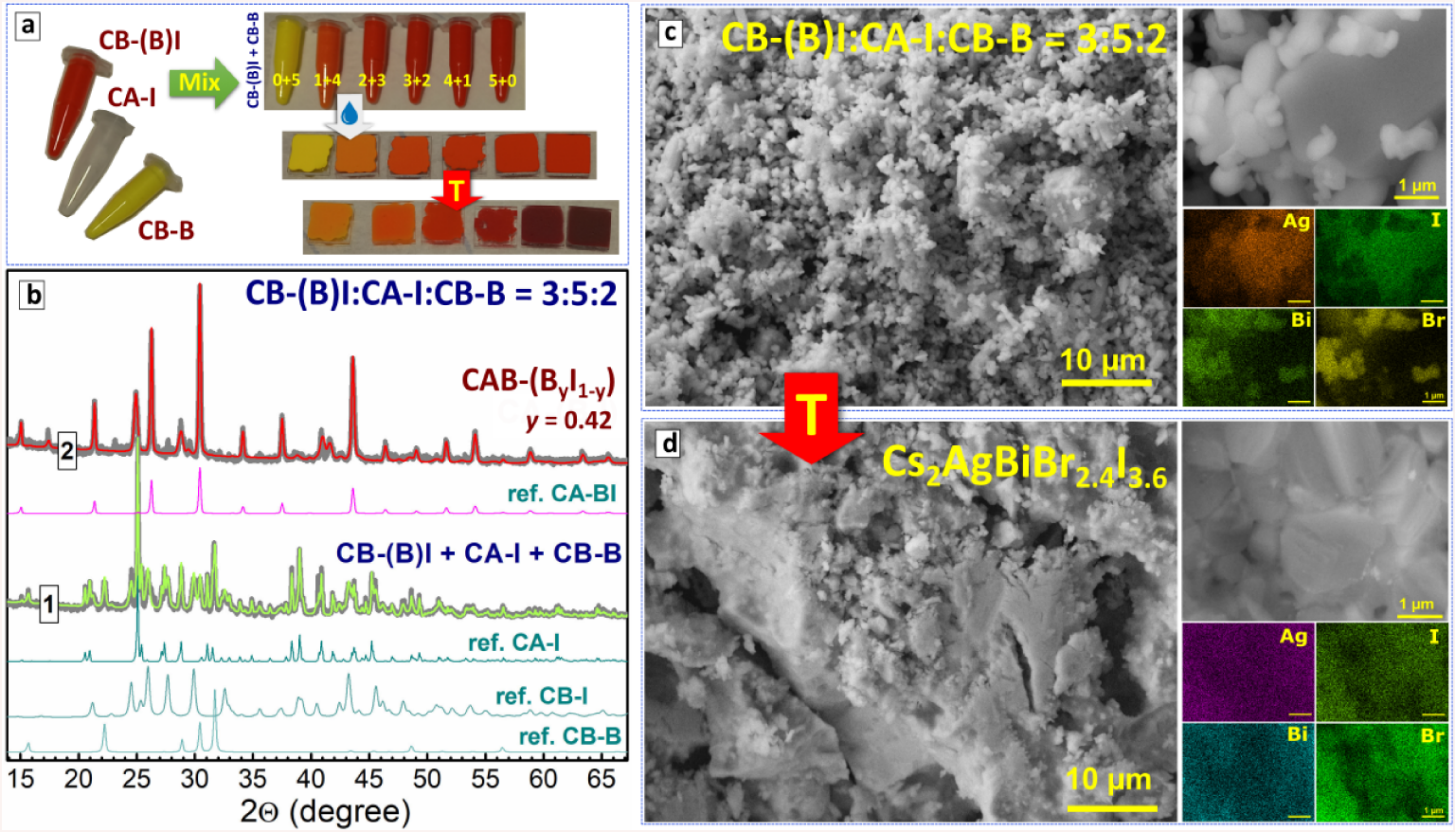
Conversion of a mixture of CB-(B)­I, CA-I, and CB-B into tetragonal CAB-(B*_y_* I_1–*y*_) perovskites: (a) schematic of the preparation of a series of CAB-(B*_y_* I_1–*y*_) perovskites with different Br/I ratios; (b) powder XRD patterns of an original mixture of double salts and final CAB-(B*_y_* I_1–*y*_) perovskite for a specific ratio of the starting precursors; (c, d) SEM images (left panels) and elemental mapping (right panels) of a mixture of CB-(B)­I, CA-I, and CB-B at a molar ratio of 3:5:2 (c) and tetragonal Cs_2_AgBiBr_2.4_I_3.6_ perovskite produced by annealing the mixture at 300 °C (d). In (b): gray lines represent experimental XRD patterns, red green/solid lines—Rietveld refinement.

According to the SEM/EDX analysis, the annealing converts the original microcrystalline mixture of double salt precursors (Figure 11c) into a compact agglomerate of submicron crystallites, showing the typical molten-lava morphology (Figure 11d). The elemental mapping of the CAB-(B_*y*_I_1–*y*_) perovskite showed a uniform distribution of Ag, Bi, Br, and I, unlike the multiphase precursors (Figure 11c), and attesting to the formation of solid-solution compounds, with variable bromide fraction and stoichiometries, typical for double perovskites (ESI, Table S4).

Upon annealing at 300 °C, the separate double salt precursors, CB-(B)­I, CB-B, and CA-I retain their morphology and spectral properties unchanged (ESI Figures S15 and S16), showing no noticeable thermal transformations. Also, no changes can be detected in PXRD patterns of binary mechanical mixtures of CB-(B)I and CB-B, subjected to annealing (ESI, Figure S17a). The thermal treatment of mixtures of CB-(B)I + CB-B with AgI yields only a small fraction of ca. 30% tetragonal perovskite product, mixed with dominant unreacted original phases (ESI, Figure S17b). The annealing of more complex mixtures of CB-(B)I + CB-B with CsI and AgI taken as 1:2 to mimic the composition of CA-I, yields a considerable fraction of ca. 60% tetragonal CAB-(B_*y*_I_1–*y*_), but still contaminated with unreacted precursors (ESI, Figure S17c). These observations further indicate the selective character of the solid-state reaction between the double salts, requiring the presence of a specific reactant, CsAg_2_I_3_, to yield tetragonal bromo-iodide perovskites. The formation of CAB-(B_*y*_I_1–*y*_) in the case of the CB-(B)I + CB-B + CsI + AgI can be assigned to the formation of a small fraction of CA-I by the contact between CsI and AgI already at RT, which can be observed spectrally by the formation of a characteristic absorption peak at ca. 330 nm, absent for pure AgI and dominating the spectrum of CA-I (ESI, Figure S18a), as well as by characteristic PXRD reflections of CsI–AgI mixtures (Figure S18b).

The variation of the CB-(B)­I/CB-B ratio in the ternary mixture of double salts is a convenient and reliable tool for producing CAB-(B_*y*_I_1–*y*_) perovskites with a tunable bromide fraction while retaining the tetragonal perovskite lattice motif. As the fraction of CB-B in the combination of CB-(BI) + CB-B increases from 0 to 100%, the PXRD reflections of CAB-(B_*y*_I_1–*y*_) gradually shift to higher angles (Figure 12a), corresponding to a contraction of the elementary cell volume from 846 Å to 736 Å (ESI, Table S4). In all cases, the products of the solid-state reaction between CB-(B)­I, CB-B, and CA-I show a characteristic tetragonal motif of the PXRD patterns (Figure 12a), a characteristic stoichiometry of double perovskites, and a mass yield of final perovskites of 85–92 wt.% (ESI, Table S4).

**Figure 12:**
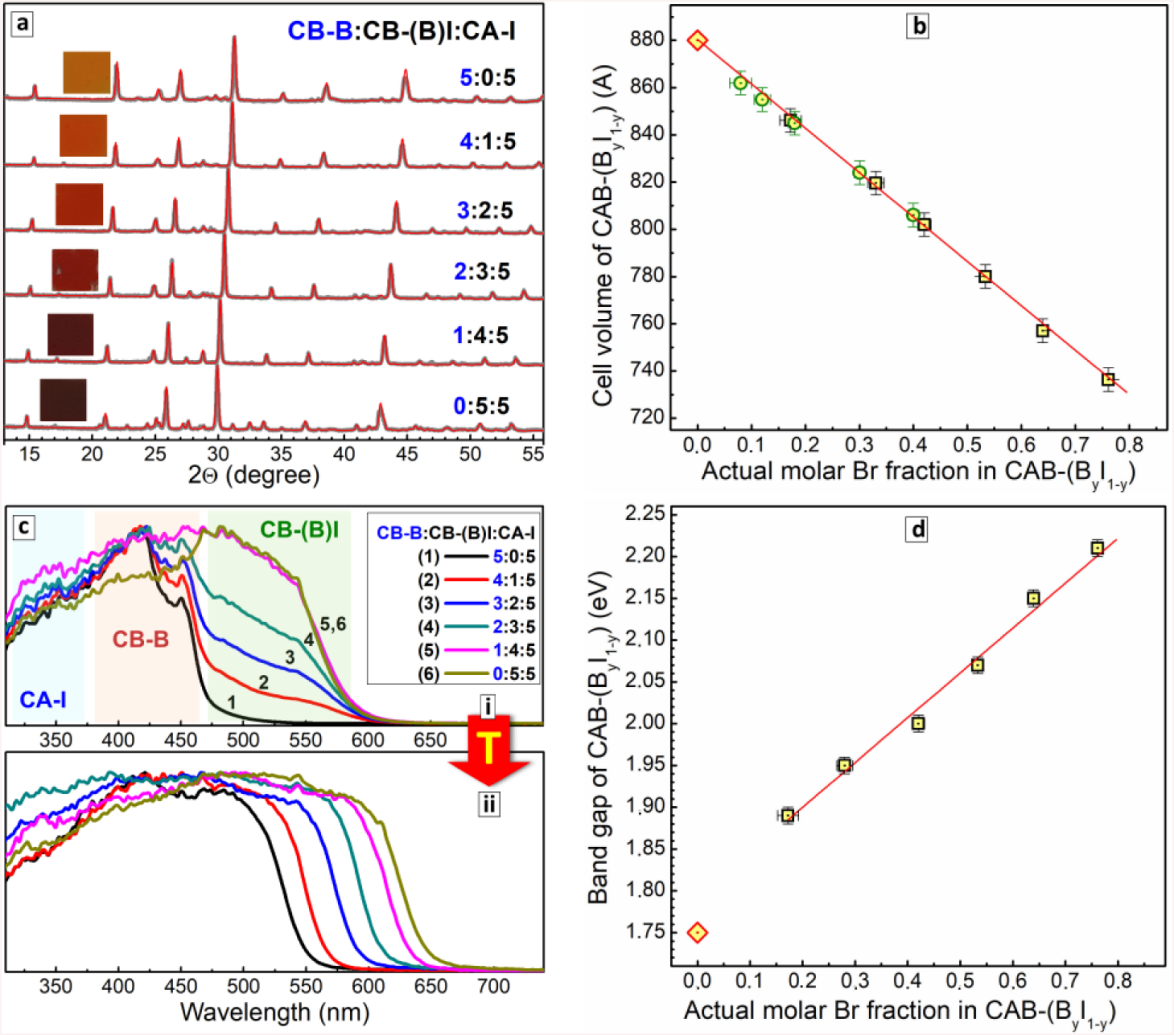
Structural and spectral data on CAB-(B*_y_* I_1–*y*_) perovskites produced by annealing of CB-B, CB-(B)­I, and CA-I double salts mixed at different molar ratios: (a) XRD patterns of the CAB-(B*_y_* I_1–*y*_) series (inserts show photographs of corresponding drop-casted films); (b) Elementary cell volume of CAB-(B*_y_* I_1–*y*_) perovskites (*V*_CAB‑(B,I)_) as a function of the actual molar bromide fraction (*v*_Br_): squares correspond to the CAB-(B*_y_* I_1–*y*_) series produced from triple mixtures of double salts, circles—to the series on different NaI amount during the anion exchange conversion of CAB-B, red diamond—data for iodide-pure tetragonal CAB-I perovskite from ref;^25^ (c) Absorption spectra of ternary CB-B, CB-(B)­I, and CA-I mixtures with different ratios (upper panel) and corresponding CAB-(B*_y_* I_1–*y*_) perovskites produced by annealing the mixtures at 300 °C; (d) Indirect bandgap of CAB-(B*_y_* I_1–*y*_) perovskites (*E*_g_) as a function of the actual molar bromide fraction, red diamond shows data for CAB-I from ref 17. Solid lines in (b) and (d) correspond to linear fits of the experimental data, yielding the following regressions: *V*_CAB‑(ByI1–y)_ = 879–186*v*_Br_, *R* ^2^ = 0.999 (b), and *E*_g_ = 1.79 + 0.54*v*_Br_, *R* ^2^ = 0.992 (d).

The elementary cell volume of CAB-(B_*y*_I_1–*y*_) perovskites increases linearly with the actual bromide fraction identified by large-area EDX analysis, encompassing the *y* range of 0.17–0.76 (Figure 12b, squares; ESI, Table S4). This dependence can be combined with a series of CAB-(B)I perovskites produced at different NaI concentrations during the AE stage and with different fractions of residual bromide (ESI, Table S1). The latter series matches the linear dependence of *V­(y)*, extending it to *y* = 0.08 (Figure 12b, squares and circles). Finally, the elementary cell volume of iodide-pure CAB-I NCs reported by D. Gamelin’s group^25^ (Figure 12b, diamond) can be added to the *V*(*y*) dependence, matching the linear character of the present data as well and complementing the range of probed bromide fractions to *y* = 0–0.76. The perfectly linear character of the relationship between the lattice cell volume and the composition of the halide sublattice provides additional evidence that all perovskite compounds summarized in Figure 12c have the same lattice type.

Observations on the spectral evolutions of the mechanical mixtures of CB-(B)­I, CB-B, and CA-I double salts taken in different proportions appeared to be as informative for tracking the thermally induced solid-state reactions as PXRD. The absorption spectra of original mixtures visualize the presence of every component: CA-I—by a characteristic peak at ca. 330 nm, CB-B—by the peak at ca. 420 nm and a shoulder at 450–460 nm, and CB-(B)­I—by the longer-wavelength contribution in the range of 470–590 nm (Figure 12c, case (i)). As the CB-(B)­I/CB-B ratio increases (from curve 1 to curves 5, 6), the features at 420 nm and 450–460 nm gradually disappear, substituted by a broad-band absorption of CB-(B)I with the edge at ca. 600 nm, allowing the ratio of both precursors to be evaluated directly from the absorption spectra.

The annealing of CB-(B)I + CB-B + CA-I mixtures at 300 °C results in the transformation of the complex absorption bands of precursors into continuous absorption bands of CAB-(B_*y*_I_1–*y*_) perovskites, showing no distinct shorter-wavelength components and a relatively sharp edge at 550–650 nm, depending on the CB-(B)­I/CB-B ratio (Figure 12c, case (ii)). The indirect band gap of CAB-(B_*y*_I_1–*y*_) calculated from the absorption slopes varies linearly with the actual bromide fraction, decreasing from 2.21 eV for *y* = 0.76 to 1.89 eV for *y* = 0.17 (Figure 12d; ESI, Table S4).

In this case, the data from Table S1 (ESI) cannot be added to *E*_g_(*y*) dependence, because the CAB-(B)­I perovskites produced at the relative NaI contents higher than 133% are contaminated with byproducts with higher band gaps. Still, the band gap of pure tetragonal CAB-I NCs reported by D. Gamelin et al.^25^ fits reasonably well into the general *E*_g_(*y*) dependence (Figure 12d, diamond). A linear fit to the present data can be extrapolated to zero bromide content, yielding the expected CAB-I band gap of 1.79 eV, close to 1.75 eV reported in ref 25. Similar to the above-discussed CAS-I perovskite, the lower *E*_g_ reported in ref 25 can be related to a stabilizing effect of ligands passivating the NC surface and retaining the tetragonal perovskite phase from the decomposition.

The linear character of band gap variation with the bromide content well corresponds to the linear dependence of the lattice cell volume on *y*, both relationships indicative of the formation of solid-solution tetragonal bromo-iodide CAB-(B_*y*_I_1–*y*_) perovskite phase with the composition of halide sublattice, reliably tunable in a broad range. Considering the general and universal character of the studied solid-state reaction between the double halide salts, more complex combinations of precursors can be envisaged, resulting in even more convoluted compositions, with simultaneous alloyings of multiple metals and halides on M^III^ and X sites.

## 6 Conclusion

An advanced approach to the mild, open-environment synthesis of the Cs_2_AgBiBr_6_ double perovskite (CAB-B) as a crystalline, single-phase product is introduced, requiring no thermal input and substantially less hydrobromic acid than synthetic protocols reported by other groups.

Anion exchange of CAB-B perovskite with NaI carried out in similarly mild conditions, yields mixtures of Cs_3_Bi_2_(Br,I)_9_ double salt with ca. 20% residual bromide (CB-(B)­I) and CsAg_2_I_3_ (CA-I), rather than the desired double iodide perovskite. However, subsequent annealing of such mixtures in the open air results in a solid-state reaction between CB-(B)I and CA-I and the formation of a tetragonal Cs_2_AgBi­(Br,I)_6_ double perovskite (CAB-(B)­I), with ca. 80% iodide in the halide sublattice. It should be noted that only a reverse reaction of the decomposition of Cs_2_AgBiI_6_ perovskite has been reported so far for nanocrystalline CAB-I destabilized by the ligand exchange.^25^ In light of the previous reports on the instability of microcrystalline CAB-I perovskite,^25,26^ the stability of the microcrystalline tetragonal CAB-(B)I perovskite reported here was attributed to the presence of residual bromide.

The feasibility of the direct solid-state reaction between CB-(B)­I and CA-I was independently proven by observing the formation of the tetragonal CAB-(B)I perovskites during the annealing of mechanical mixtures of separately produced CB-(B)I and CA-I, as well as mixtures of anion-exchanged CAB-B with additional amounts of CA-I.

A multiparameter optimization of the two-stage synthesis of CAB-(B)­I perovskites was performed to identify the optimal conditions for both the first-stage room-temperature anion exchange and the second-stage annealing, thereby achieving the highest yields of the tetragonal CAB-BI phase with the lowest band gaps. At that, the optimal amount of NaI excess, water/2-propanol ratio in the solvent, and the durations of the contact between CAB-B and NaI were found in rather narrow ranges, reflecting a delicate balance between the depth of the bromide-to-iodide exchange and inhibition of the solid-state reaction between CB-(B)­I and CA-I by water-induced hydrolytic passivation. Variations of the duration and temperature of the annealing additionally attested to a remarkable thermal stability of the tetragonal CAB-(B)I perovskites and showed that the highest yields of this phase, 85–92 wt.%, the largest volume of the elementary cell indicative of the highest iodide content of 88%, and the band gap slightly below 1.9 eV can be achieved by performing the solid-state reaction at 290–300 °C.

By modifying the basic AE/T process with additional steps of manual grinding and a second annealing of the CAB-(B)I perovskite, the yield of the tetragonal perovskite can be elevated above 99 mass%, thereby demonstrating the feasibility of synthesizing phase-pure perovskite products within the proposed two-stage approach. Moreover, it can be generalized to a broad range of halide precursors at both stages (anion exchange and solid-state reaction), thereby significantly increasing the complexity of the final tetragonal perovskite products.

The potential for generalization can be realized by introducing more complex halide precursors into the RT AE or by using more complex mixtures of halide double salts in solid-state reactions, thereby enabling the production of solid-solution tetragonal perovskites via two alternative pathways, as illustrated in Figure 3.

The applicability of the basic RT AE/T process to more complex perovskite precursors was demonstrated by the two-stage conversion of Cs_2_AgBi_*x*_Sb_1–*x*_Br_6_ (CABS-B) precursors into tetragonal Cs_2_AgBi_*x*_Sb_1–*x*_(Br,I)_6_ perovskites (CABS-(B)­I), which exhibit band gaps lower than those of the corresponding Bi-only counterparts due to the band-bowing effect. In the optimized conditions, the CABS-(B)­I perovskite with the highest iodide content of ca. 90% and the lowest indirect band gap of 1.78 eV was produced at the annealing temperature of 300 °C and the Bi/Sb ratio of 1:1. The same CABS-(B)I perovskites with variable bismuth/antimony ratios can alternatively be formed by thermal solid-state reactions between halide double salts in ternary mechanical mixtures of CA-I, CB-(B)­I, and CS-I, the latter two precursors produced from corresponding bromide and chloride double salts via RT AE.

The solid-state reactions among ternary and potentially more complex mixtures of double salts can be broadly applied to achieve controlled alloying in both cationic and anionic sublattices. Along with the above-discussed Bi/Sb solid-solutions, alloyed bromo-iodide tetragonal Cs_2_AgBi­(Br_*y*_I_1–*y*_) perovskites (CAB-(B_*y*_I_1–*y*_)) with high yields (85 wt.% and higher) and a variable *y* were produced in solid-state reactions between CB-(B)­I, CA-I, and CB-B double salts, combined in specific ratios. The CAB-(B_*y*_I_1–*y*_) perovskites showed ideal solid-solution compositional variation of the lattice parameters and band gaps in a broad range of *y* = 0.08–0.76.

In summary, the two-stage RT AE/T approach developed in the present work provides a general, flexible, and sustainable pathway for the combinatorial synthesis of stable and phase-pure tetragonal double perovskites with broadly variable compositions. It shows significant potential for further increasing compositional complexity through independent design of both stages and the introduction of more complex, compositionally versatile metal-halide precursors.

## Supplementary Material



## Notes

Materials and methods; list of compositional abbreviations; additional experimental data on SEM, EDX, PXRD, and spectral characterizations of metal-halide precursors; intermediate products of the anion exchange; final tetragonal perovskite products (PDF)
