# Supplementary material for: Lead-Free Double Cs2Ag(Bi,Sb)(Br,I)6 Perovskites: Going below 1.8 eV Bandgap by Anion Exchange and Solid-State Reactions
Source: J Am Chem Soc. 2026 May 18;148(21):22364–79. doi: 10.1021/jacs.6c07190 (PMC13244445; doi:10.1021/jacs.6c07190)
Supplement: Supplementary file 1 [file ja6c07190_si_001.pdf]

Electronic Supporting Information

Lead-free Double $Cs_2Ag(Bi,Sb)(Br,I)_6$ Perovskites: Going Below 1.8-eV Bandgap by Anion Exchange and Solid-State Reactions

Oleksandr Stroyuk[1*], Oleksandra Raievska[1],
Sachin Kinge[2],
Jens Hauch[1,3], Christoph J. Brabec[1,3]

[1]*Forschungszentrum Jülich GmbH, Helmholtz-Institut Erlangen-Nürnberg für Erneuerbare Energien (HI ERN), 91058 Erlangen, Germany*
[2]*Materials Engineering Div., Toyota Motors Europe, sachin.kinge@toyota-europe.com*
[3]*Friedrich-Alexander-Universität Erlangen-Nürnberg, Materials for Electronics and Energy Technology (i-MEET), Martensstrasse 7, 91058 Erlangen, Germany*

Authors for correspondence:

[*]Dr. Oleksandr Stroyuk, Forschungszentrum Jülich GmbH, Helmholtz-Institut Erlangen-Nürnberg für Erneuerbare Energien (HI ERN), Immerwahrstr. 2, 91058 Erlangen, Germany; *e-mail*: o.stroyuk@fz-juelich.de, alstroyuk@ukr.net.

Materials and Methods

I. Preparation of stock solutions

(1) 5.0 M HBr in H_2O: 5.6 mL of concentrated (8.9 M, 48 w.%) HBr is slowly added to 4.4 mL of deionized (DI) water under intense refluxing. *Note*: all operations with HBr solutions should be performed only under a fume hood.

(2) 1.0 M $BiBr_3$ in 5.0 M aqueous HBr: 4.487 g of $BiBr_3$ is added to 9.0 mL of 5.0 M aqueous HBr solution; after the complete dissolution of bismuth bromide, 5.0 M solution of HBr is added till the total solution volume of 10.0 mL is achieved.

(3) 1.0 M $SbBr_3$ in 5.0 M aqueous HBr: 3.615 g of $SbBr_3$ is added to 9.0 mL of 5.0 M aqueous HBr; after the complete dissolution of antimony bromide, 5.0 M solution of HBr is added till the total solution volume of 10.0 mL is achieved.

(4) 1.0 M $AgNO_3$ in H_2O: 1.699 g of $AgNO_3$ is added to 9.0 mL of DI water; after the complete dissolution of silver nitrate, additional water is added till the total solution volume of 10.0 mL is achieved.

(5) 4.0 M CsAc in H_2O: 7.678 g of cesium acetate (CsAc) is added to 6.0 mL of DI water; after the complete dissolution of CsAc, additional water is added till the total solution volume of 10.0 mL is achieved.
(6) 4.0 M NaI in H_2O: 5.996 g of NaI is added to 6.0 mL of DI water; after the complete dissolution of NaI, additional water is added till the total solution volume of 10.0 mL is achieved.

II. Synthesis of microcrystalline $Cs_2AgBi_xSb_{1-x}Br_6$ (CABS-B)

Microcrystalline CABS-B samples are produced from two separately prepared precursor solutions mixed at intense refluxing at room temperature (RT) in open-atmosphere conditions. Precursor I was produced by mixing x mL of 1.0 M $BiBr_3$ solution in 5.0 M aqueous HBr, (1-x) mL of 1.0 M $SbCl_3$ solution in 5.0 M aqueous HBr, 1.0 mL of 8.9 M aqueous HBr, and 5.0 mL 2-propanol under magnetic refluxing. Precursor II was prepared by mixing 1.0 mL of 1.0 M aqueous $AgNO_3$ solution, 1.2 mL DI water, 0.25 mL of 25 w.% aqueous NH_4OH solution, 0.55 mL of 4.0 M aqueous CsAc solution, and 5.0 mL of 2-propanol. It should be noted that precursor II contains 10 molar% excess of CsAc with respect to the expected stoichiometry to ensure complete precipitation of the final perovskite products and suppress the formation of double $Cs_3(Bi, Sb)_2Br_9$ salts. The excess CsAc remains dissolved in the supernatant after precipitation and can be separated from the products during purification. All components of Precursor II are mixed under magnetic refluxing.

Precursor I is placed in a glass 25-mL vial, and Precursor II is rapidly added under intense magnetic refluxing. The resulting suspension is refluxed under an open atmosphere at RT for 5 min, then the vial is capped with a Parafilm layer and left overnight without refluxing. Afterward, the precipitate is refluxed again to form a homogeneous suspension, which is transferred into a 15-mL centrifuge vial, capped, and centrifuged at 1500 rpm for 2 min. Subsequently, the supernatant is separated, and the microcrystalline precipitate undergoes multiple purification steps. For this, 10.0 mL of 2-propanol is added to the precipitate; the mixture is refluxed to restore homogeneity, then centrifuged to separate the supernatant. The purification is repeated, yielding final microcrystalline products that are left in a Petri dish in the air (20-25 ^oC, relative humidity of 40-50%) for solvent evaporation, then placed in closed vials and stored in the dark under ambient conditions.

III. Post-synthesis mechanical grinding/annealing of CAB-(B)I perovskite

In a typical procedure, 0.1 mol (0.1960 g) of CB-(B)I and 0.1 mol (0.0729 g) CA-I were loaded into an agate mortar, 0.5 mL of anhydrous 2-propanol was added, and the suspension was manually ground for 5 min at RH of 30% and 20 ^oC. The ground suspension was drop-cast on a glass substrate, left for solvent evaporation for 10 min, and annealed at 300 ^oC for 10 min in open air at the same environmental conditions.

The annealed product was detached from the glass, placed into the agate mortar, 0.5 mL of anhydrous 2-propanol was added, and the suspension was subjected to the second grinding in the same conditions. Then, the suspension was again drop-cast onto the glass substrate and subjected to the second annealing in air at 300 ^oC for 10 min.

As an alternative to manual grinding, the t-CAB-(B)I produced by the first annealing was detached from the glass substrate, placed into an 80-mL zirconia vessel, mixed with 25 2-mm zirconia balls, and subjected to a mechanochemical treatment in a planetary ball mill at 500 rpm for 1 h.

IV. Synthesis of microcrystalline $Cs_2Ag(Bi, Sb)(Br, I)_6$ samples (CABS-(B)I) by basic RT AE/T process

The above-described CABS-B samples are brought into contact with water/2-propanol solutions of NaI for a fixed period under intense reflux. This procedure results in partial anion exchange (AE) in CABS-B and the formation of an intermediary product, denoted AE-CABS-B. This intermediate is purified similarly to the method described above for CABS-B samples to remove NaBr formed during anion exchange. In the second step, AE-CABS-B products undergo thermal annealing in air to form the final CABS-BI perovskites.

In a typical procedure, 1 mmol of dry microcrystalline CABS-B is placed in a 25-mL glass vial, 5.0 mL of anhydrous 2-propanol is added, the vial is capped with a Parafilm seal, and the mixture is subjected to intense magnetic stirring under reflux for 15 min to form a homogeneous suspension. Then, an AE solution is added under intense reflux, containing 10 mL of 2-propanol and 2.0 mL of 4.0 M aqueous NaI solution. In these conditions, the AE solution contains a 33% excess of NaI with respect to the amount stoichiometrically necessary for the complete substitution of the present bromide anions, or 133% with respect to the present bromide. The presence of excessive NaI shifts the equilibrium toward the formation of the iodide phase and increases the fraction of iodide in the final products.

The mixture is left under reflux for 20 min, then transferred to a 50-mL centrifuge vial and centrifuged at 1500 rpm for 2 min. Afterward, the supernatant is completely removed, 10.0 mL of 2-propanol is added, and the mixture is stirred until a homogeneous suspension forms. This suspension is purified as discussed above in section II, with the purification step repeated twice. The final precipitate is left in open air at RT to allow solvent evaporation and then stored in closed vials under ambient conditions in the dark.

The as-prepared AE-CABS-B products are annealed in air. For this, the microcrystalline AE-CABS-B is distributed as a thin uniform layer on a glass plate or a Petri dish and calcined on an open-air heating plate at 290 ^oC for 10 min. The products are left to cool naturally, transferred into closed vials, and stored in the dark under ambient conditions. In series with varied annealing temperature or annealing duration, 50 mg of dry AE-CABS-B was mixed with 200 µL of 2-propanol, and refluxed to form a homogeneous suspension. The suspension was then drop-cast onto a series of glass substrates (40 µL per 1 cm^2 plate), left under ambient conditions to allow solvent evaporation, and annealed for specific periods or at specified temperatures in air.

V. Synthesis of microcrystalline $Cs_3(Bi_xSb_{1-x})_2Br_9$ (CBS-B)

Microcrystalline CBS-B was produced from two separately prepared precursors, which were mixed under intense reflux in an open atmosphere at RT. Precursor I was produced by mixing x mL of 1.0 M $BiBr_3$ in 5.0 M aqueous HBr, (2-x) mL of 1.0 M $SbBr_3$ in 5.0 M aqueous HBr, 2.0 mL of 8.9 M (48 w.%)

aqueous HBr, and 5.0 mL of 2-propanol. The Bi fraction was varied over $0 < x < 2$, and all components were added under intense magnetic reflux. Precursor II was prepared by mixing 0.75 mL of DI H_2O, 0.75 mL of 4.0 M aqueous CsAc solution, and 5.0 mL of 2-propanol under intense magnetic refluxing. Precursor I is placed in a 25-mL glass vial, and Precursor II is rapidly added under magnetic stirring, forming a suspension that is refluxed for 5 min, then sealed with Parafilm and left unstirred overnight.

Afterward, 2 mL of the supernatant is separated and discarded, while the remaining maternal solution and precipitate are transferred to a 15-mL centrifuge vial and centrifuged at 1500 rpm for 2 min. Then, the supernatant is completely removed, and 10.0 mL of 2-propanol is added. This mixture is refluxed until a homogeneous suspension forms, then centrifuged. Such a purification procedure is repeated. The final precipitate is left to dry at 20-25 ^{o}C and 40-50% RH in a Petri dish, transferred into a closed vial, and stored in the dark under ambient conditions.

VI. Synthesis of microcrystalline $Cs_3(Bi,Sb)_2(Br,I)_9$ (CBS-BI)

Microcrystalline CBS-BI samples were produced by anion exchange of the above-described CBS-B series with water/2-propanol solution of sodium iodide, followed by purification from NaBr product. In a typical procedure, 1 mmol of dry microcrystalline CBS-B was placed in a 25-mL glass vial, 5.0 mL of 2-propanol was added, the vial was sealed with Parafilm, and the mixture was refluxed for 15 min to form a homogeneous suspension. Subsequently, a mixture of 10 mL of 2-propanol and 2.25 mL of 4.0 M aqueous NaI solution is added under intense magnetic reflux, and the suspension is refluxed for 30 min. Afterward, the mixture was transferred to a 50-mL centrifuge vial and centrifuged at 1500 rpm for 2 min. The supernatant was completely removed, and 10.0 mL of 2-propanol was added. The purification procedure was then performed twice, as discussed above. The final precipitate was left in an open Petri dish at RT to allow solvent evaporation, then transferred to closed vials and stored in the dark under ambient conditions.

VII. Synthesis of microcrystalline $CsAg_2I_3$ (CA-I)

Microcrystalline CA-I was produced following an original approach, from two precursors, which were mixed under intense magnetic reflux. Precursor I was prepared by mixing 2.0 mL of 1.0 M aqueous $AgNO_3$ solution, 0.6 mL of 25 wt.% aqueous NH_4OH solution, and 0.3 mL of 4.0 M aqueous CsAc solution. Precursor II was prepared by mixing 2.0 mL of 4.0 M aqueous NaI solution, 1.0 mL DI H_2O, and 30 mL of 2-propanol. All additions were performed under intense magnetic refluxing.

Precursor I was placed in a 50-mL glass vial, and Precursor II was added under intense refluxing. The mixture was refluxed for 5 min, capped with a Parafilm layer, and left unstirred overnight in the dark. Subsequently, the mixture was refluxed to rehomogenize it, then transferred to a 50-mL centrifuge vial and centrifuged at 1500 rpm for 2 min. Then, the supernatant is completely removed and discarded, and 10 mL of 2-propanol is added. The purification is performed twice, as discussed above. The final precipitate was left in an open Petri dish at RT to allow solvent evaporation, then transferred to closed vials and stored in the dark under ambient conditions.

VIII. Preparation of samples for characterization

In a typical preparation for spectral and powder XRD measurements, 50 mg of dry powder was mixed with 200 µL of 2-propanol, refluxed until a homogeneous suspension formed, drop-cast onto a glass substrate (40 µL per 1 cm^2), and left to evaporate the solvent under ambient conditions. For SEM/EDX measurements, 1 µL of sample suspension was drop-cast on 1 mm^2 of double-sided carbon adhesive tape mounted on a silicon wafer.

IX. Characterizations

Powder XRD patterns were registered using a Panalytical X'pert powder diffractometer with filtered Cu K_α radiation (λ = 1.54178 Å) and an X'Celerator solid-state stripe detector in the Bragg-Brentano geometry in an angle range of 2θ = 5-100° with a step rate of 0.05° per min. The XRD patterns were subjected to Rietveld refinement using MAUD (version 2.99) with structural CIF files from the Crystallography Open Database (https://www.crystallography.net/cod/). In all examinations of annealed products, the samples were collected after annealing was completed, cooled to RT, and subjected to PXRD measurements under ambient conditions.

COD ID numbers for the CIF files used in the refinements are 1011025 (AgI), 1509151 (AgBr), 2106275 (hexagonal $Cs_3Bi_2I_9$), 1537138 (trigonal $Cs_3Sb_2Br_9$), 1520793 (trigonal $Cs_3Sb_2I_9$), 4131244 (cubic $Cs_2AgBiBr_6$), and 2310071 ($CsAg_2I_3$).

SEM imaging and EDX analysis were performed using a JEOL JSM-7610F Schottky field emission scanning electron microscope operating under 15-20 kV acceleration voltage and equipped with an X-Max 80 mm^2 silicon drift detector (Oxford Instruments) and AZtec nanoanalysis software. For each sample, EDX spectra were collected from at least three different spots, each ca. 10×10 µm in area, as well as from a panoramic area of ca. 100×100 µm, and the results were averaged. In selected experiments with samples containing two phases with different morphologies, EDX spectra were collected for each morphology in at least three manually selected areas where the target morphology was prevalent, and the calculated atomic fractions were averaged for each morphology.

Reflectance spectra were recorded using a BlackComet spectrometer (StellarNet Inc.) and a 75-W Xenon lamp (Thorlabs) as an excitation source. The spectra were recorded using an optical Y-fiber probe in an identical geometry for both the samples and a scattering reference (ultra-pure $BaSO_4$, Alfa Aesar). The reflectance spectra were converted to absorption spectra using the Kubelka-Munk formula and a reference.

List of abbreviated names and corresponding formulas

CAB-B	$Cs_2AgBiBr_6$
CABS-B	$Cs_2Ag(Bi,Sb)Br_6$
CAB-(B)I	$Cs_2AgBi(Br,I)_6$
CAB-(B_yI_{1-y})	$Cs_2AgBi(Br_yI_{1-y})_6$
CABS-(B)I	$Cs_2Ag(Bi,Sb)(Br,I)_6$
CB-B	$Cs_3Bi_2Br_9$
CS-C	$Cs_3Sb_2Cl_9$
CS-I	$Cs_3Sb_2I_9$
CBS-B	$Cs_3(Bi,Sb)_2Br_9$
CB-(B)I	$Cs_3Bi_2(Br,I)_9$
CBS-(B)I	$Cs_3(Bi,Sb)_2(Br,I)_9$
CA-I	$CsAg_2I_3$
AE-CAB-B	non-annealed products of anion exchange of CAB-B with NaI at RT
AE-CABS-B	non-annealed products of anion exchange of CABS-B with NaI at RT

Figures

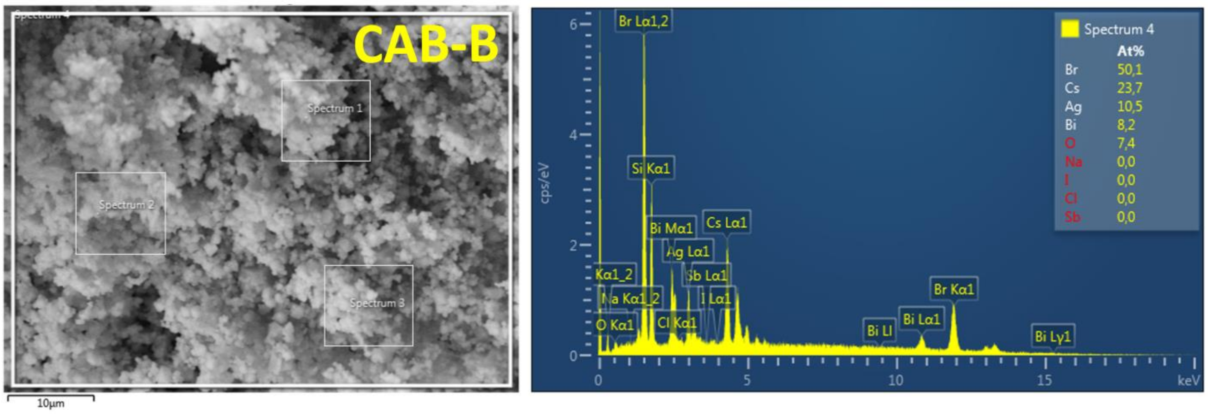

Figure S1. SEM image of cubic CAB-B perovskite showing four selected areas used for EDX analysis (spots of ca. 10×10 μm for spectra 1-3 and a panoramic spot of ca. 100×100 μm for spectrum 4) with an exemplary panoramic EDX spectrum.

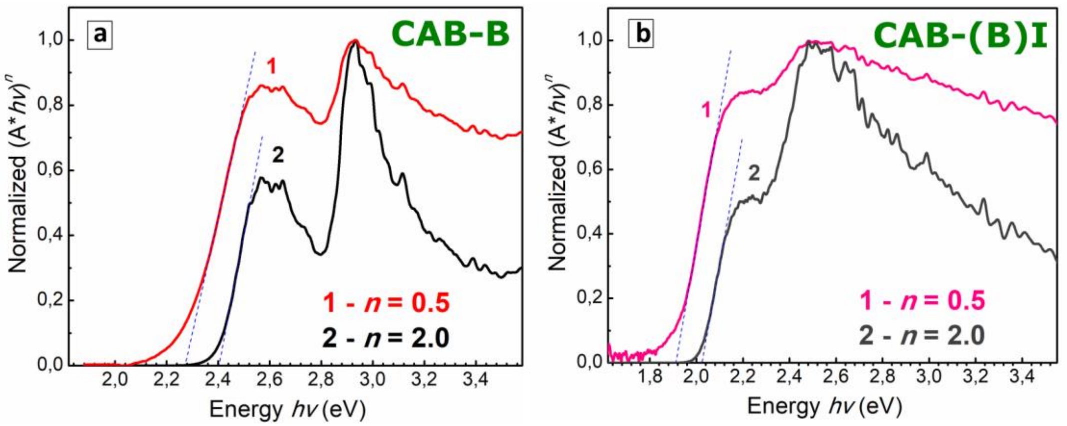

Figure S2. Normalized absorption spectrum of CAB-B (a) and CAB-(B)I (b) perovskites presented in Tauc coordinates for indirect (curve 1, n = 0.5) and direct (curve 2, n = 2.0) allowed electronic transitions.

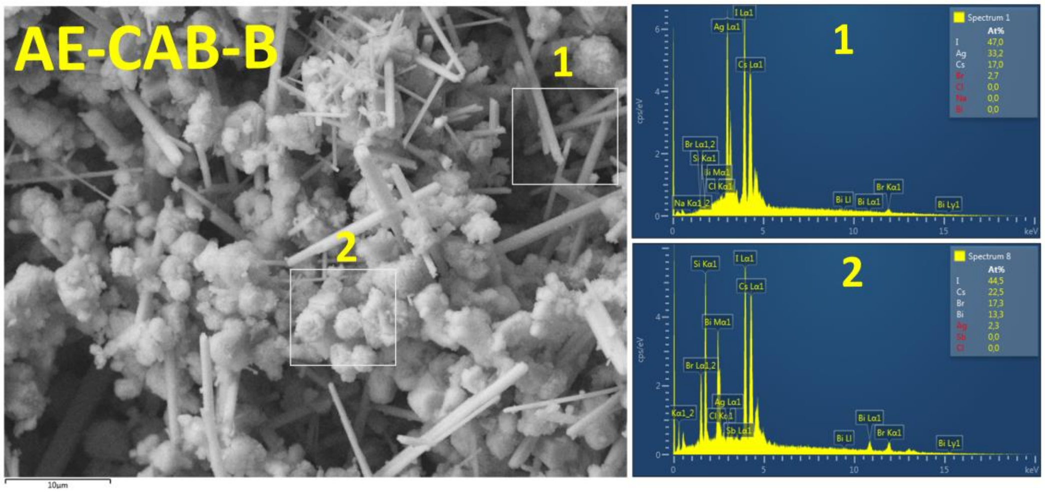

Figure S3. SEM image of a mixture of CB-(B)I and CA-I double salts produced by the anion-exchange-driven conversion of CAB-B perovskite (AE-CAB-B), showing two selected areas used for EDX analysis of different phases and morphologies with corresponding EDX spectra and calculated atomic fractions.

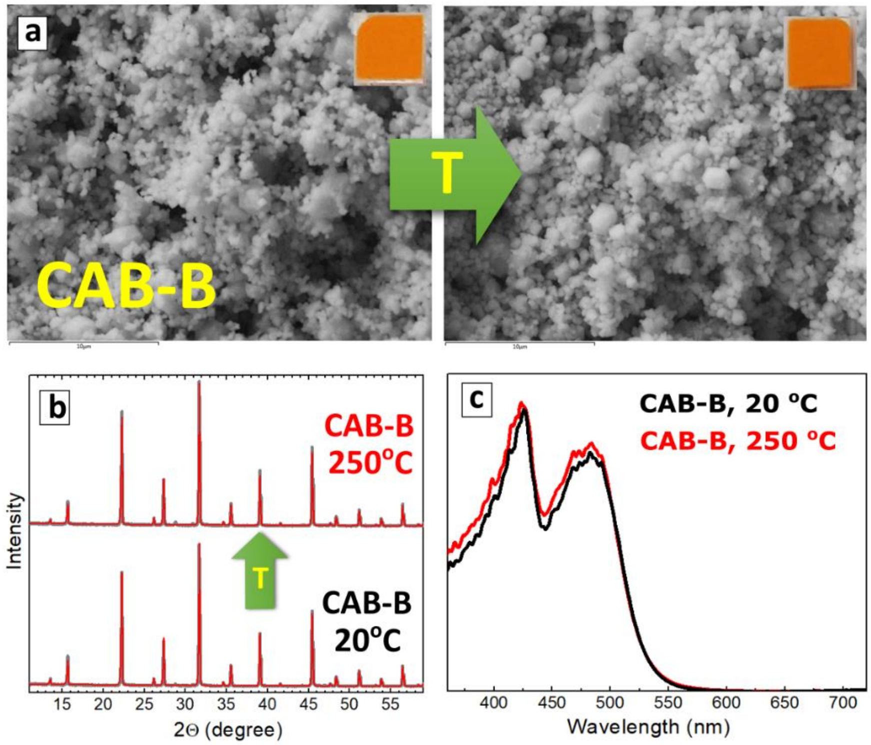

Figure S4. SEM images (a), powder XRD profiles (b), and absorption spectra (c) of CAB-B perovskites before and after the annealing in air at 250 °C. Inserts in (a) show photographs of corresponding samples drop-casted on glass. In (b), the gray and red lines show experimental data and Rietveld refinement, respectively.

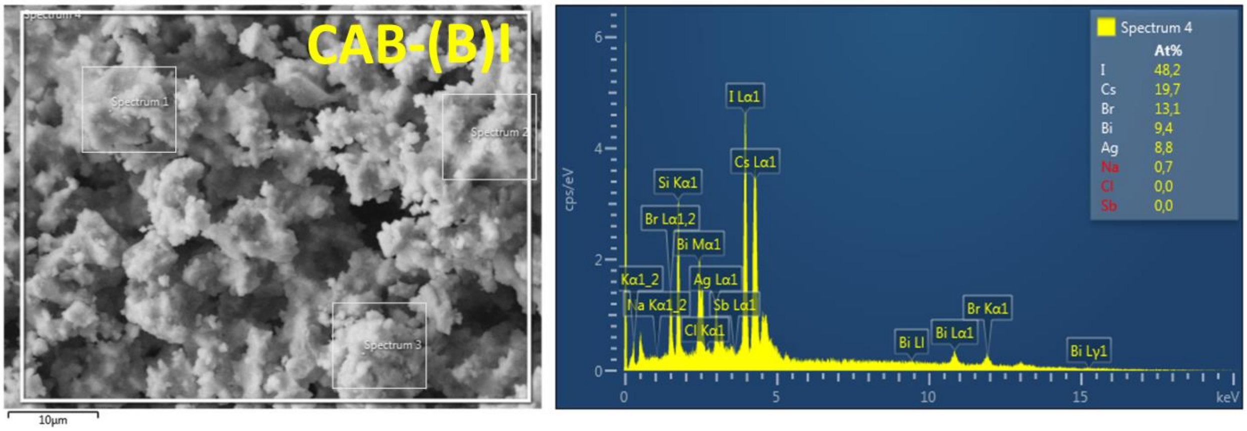

Figure S5. SEM image of tetragonal CAB-(B)I showing four selected areas used for EDX analysis (spots of ca. 10×10 μm for spectra 1-3 and a panoramic spot of ca. 100×100 μm for spectrum 4) with an exemplary panoramic EDX spectrum.

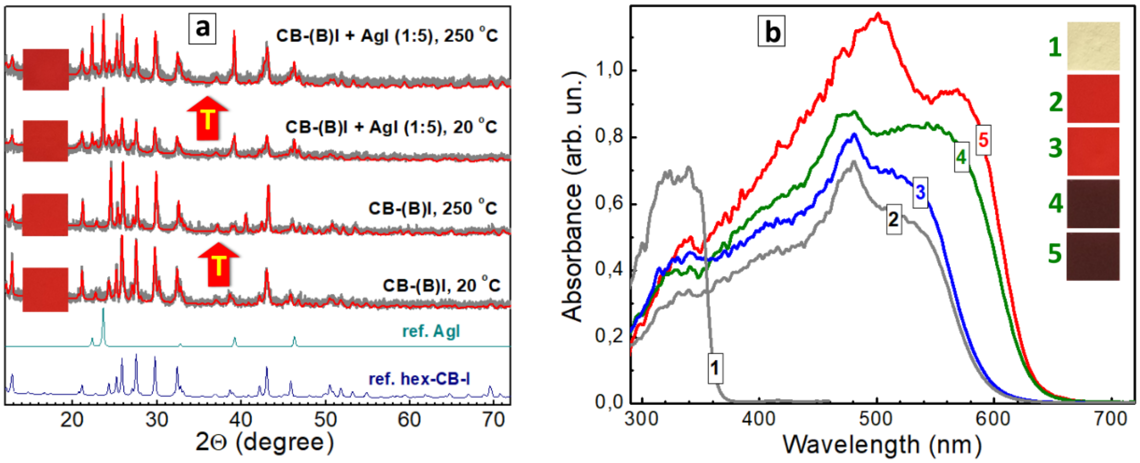

Figure S6. (a) Powder X-ray profiles for CB-(B)I and a mixture of CB-(B)I+AgI kept at RT and annealed in air at 250 °C. The insert shows photographs of the corresponding samples. Gray lines correspond to experimental data, red lines – Rietveld refinements. (b) Absorption spectra of CA-I (curve 1), CB-(B)I (2), a mechanical mixture of CA-I and CB-(B)I, 1:1 (3), products of CB-(B)I+CA-I annealing at 250 °C (4), and CAB-(B)I perovskite produced by AE-CAB-B annealing as a reference (5).

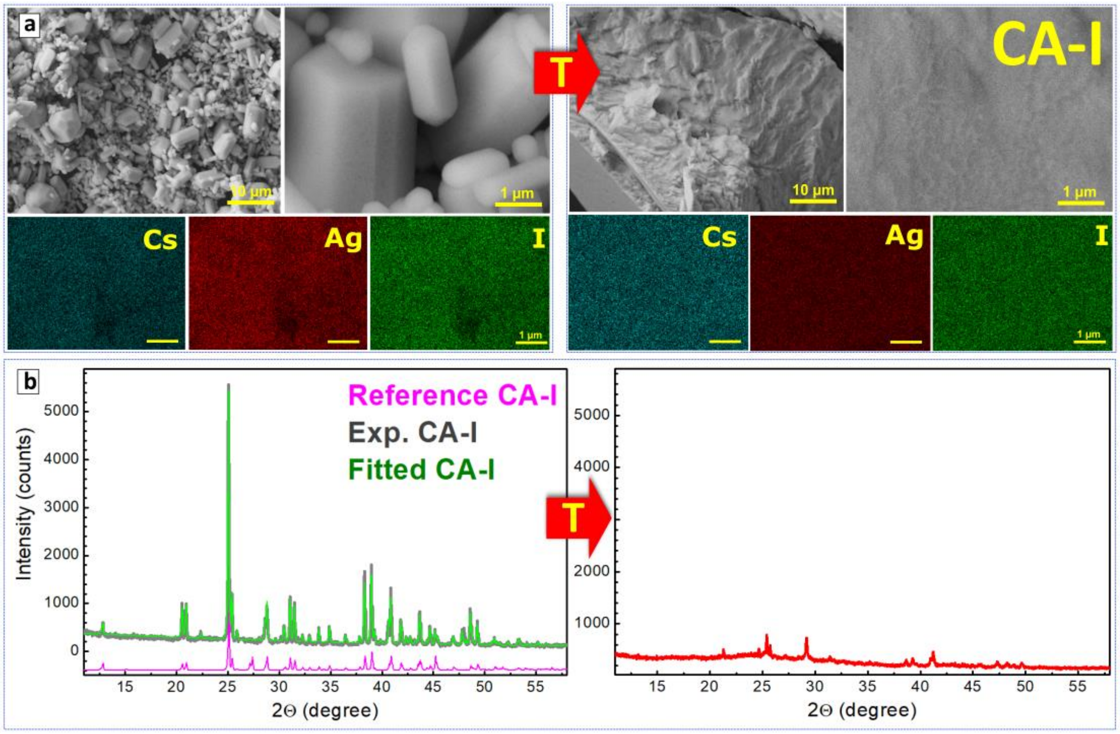

Figure S7. SEM images, element distribution maps (a), and powder XRD patterns (b) of microcrystalline CA-I double salt directly after the synthesis (left panels) and after the open-air annealing at 250 °C (right panels).

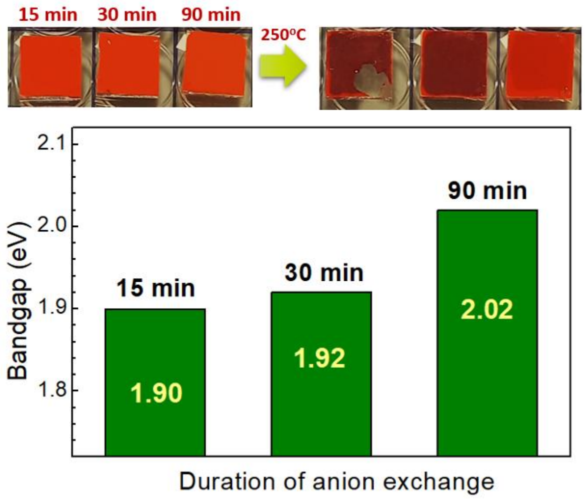

Figure S8. Bandgap of CAB-BI produced at different contact times between CAB-B and a solution of NaI in water/IP (120% NaI, water fraction of 0.15). The upper panel shows photographs of drop-casted samples produced at different contact times with NaI before and after the annealing at 250 °C for 10 min.

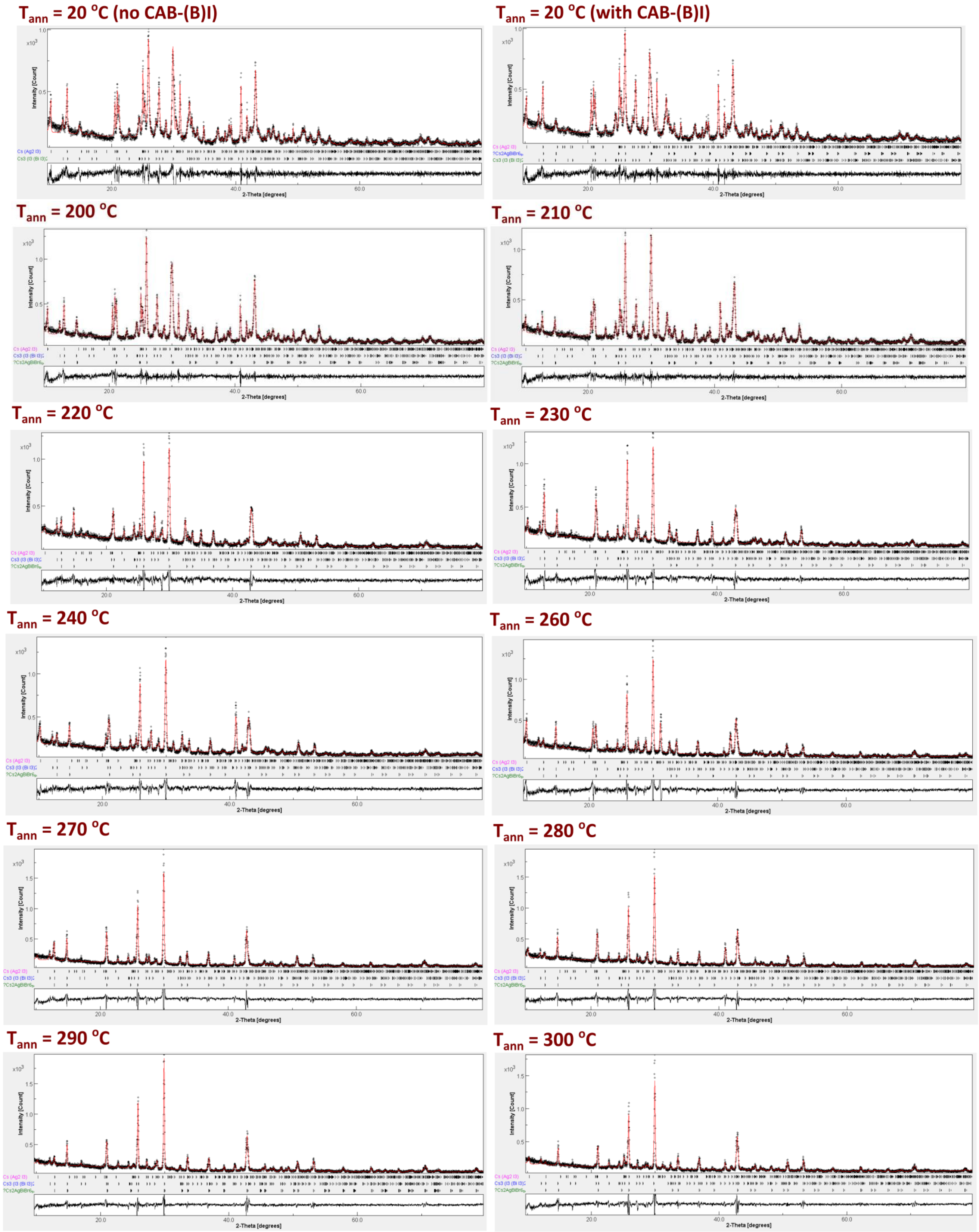

Figure S9. A collection of screenshots of MAUD-processed Rietveld refinement of a series of CAB-(B)I samples produced at different annealing temperatures, T_{ann}. For T_{ann} = 20 °C, two cases of fitting are presented, corresponding to phase mixtures without and with CAB(B)I admixture.

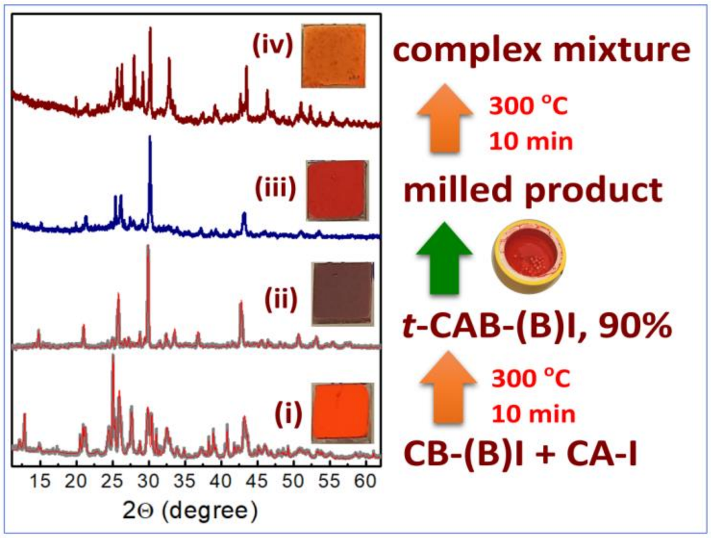

Figure S10. Powder XRD patterns of a starting mixture of CB-(B)I and CA-I double salts (i), tetragonal *t*-CAB-(B)I perovskite formed after the first annealing of such mixture at 300 ^{o}C for 10 min (ii), products of a mechanochemical treatment in a planetary ball mill (iii), and annealing of the ball-miling products at 300 ^{o}C for 10 min (iv). The schematic on the right panel illustrates the sequence of treatments.

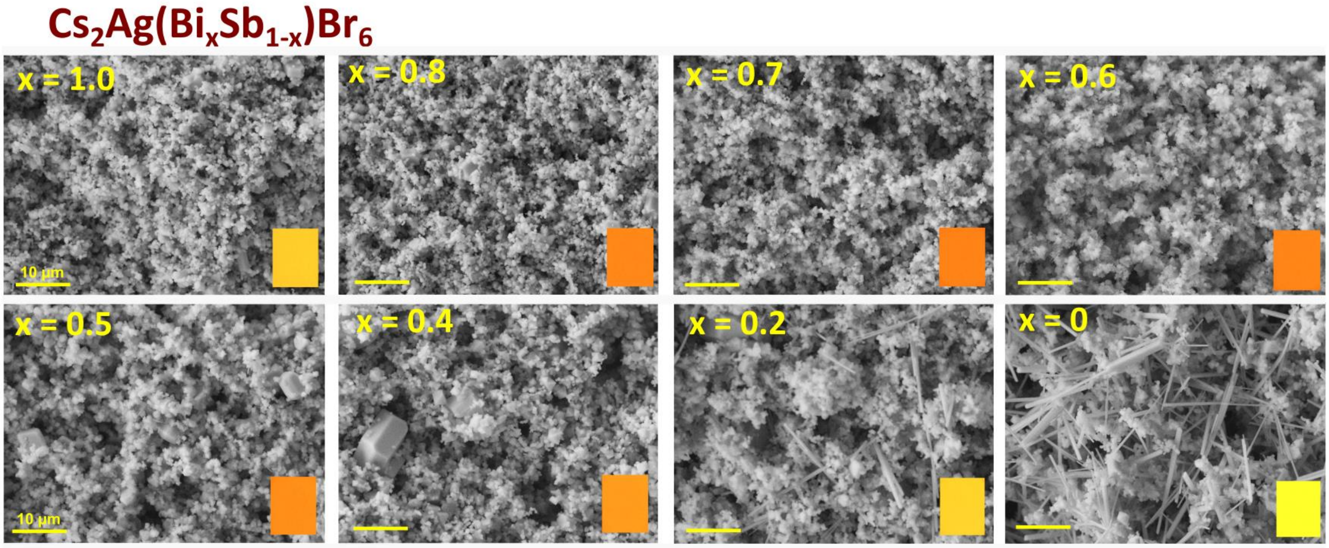

Figure S11. SEM images of $Cs_2Ag(Bi_xSb_{1-x})Br_6$ samples with varied nominal Bi fraction x. Inserts show photographs of corresponding samples as drop-cast films on glass.

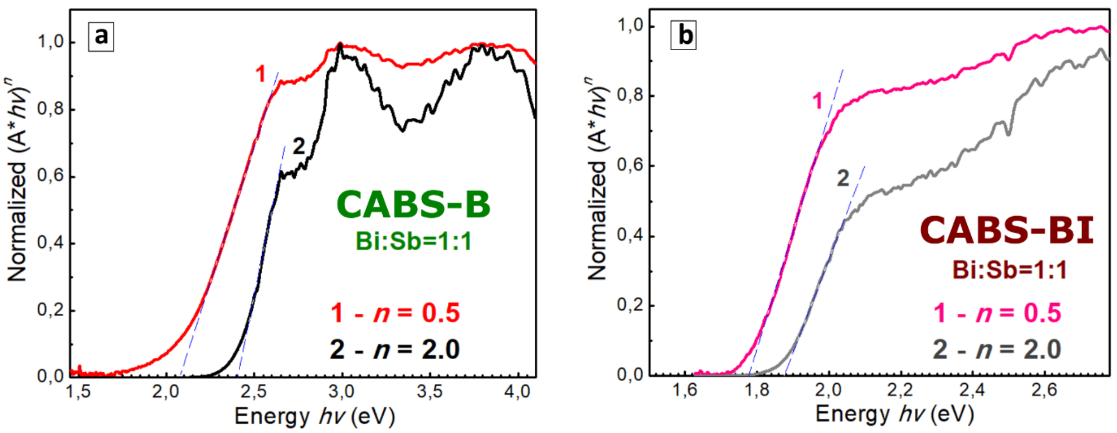

Figure S12. Normalized absorption spectrum of CABS-B (a) and CABS-BI (b) perovskites presented in Tauc coordinates for indirect (curve 1, n = 0.5) and direct (curve 2, n = 2.0) allowed electronic transitions. Bi:Sb = 1:1.

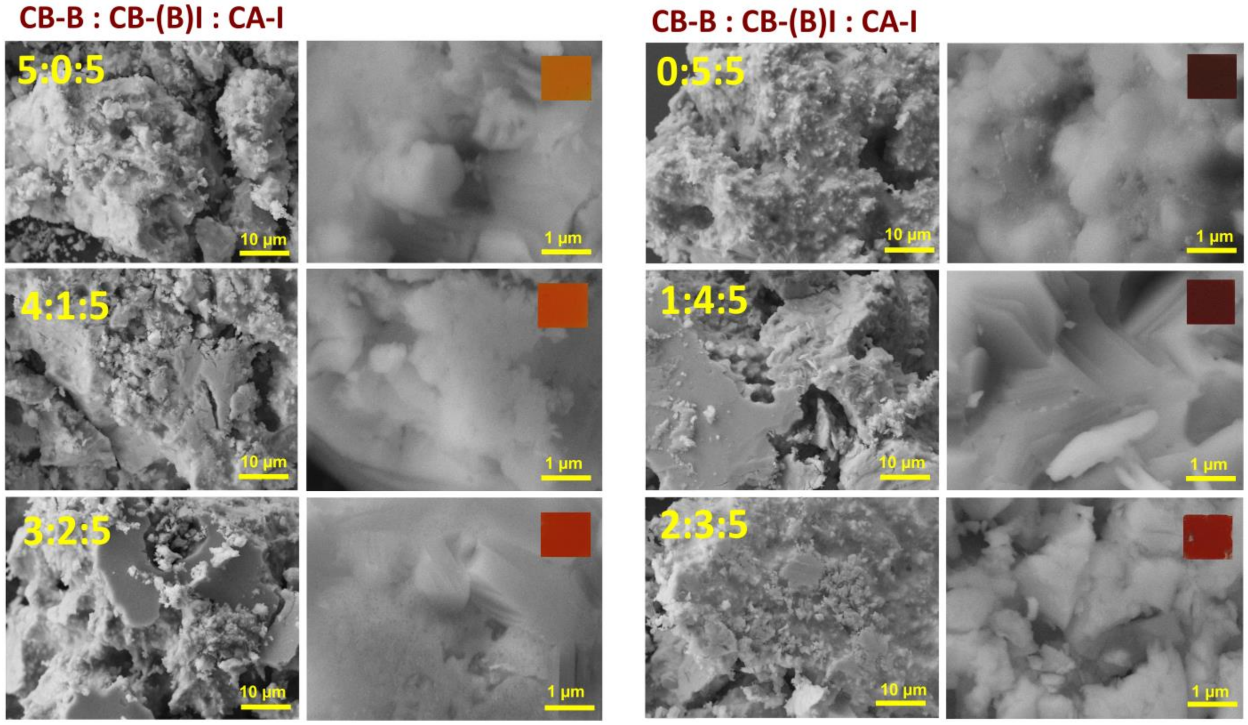

Figure S13. Collection of SEM images of CAB-(B,I) perovskites produced by annealing of the ternary CB-B, CB-(B)I, and CA-I double salts mixed in different molar ratios (indicated on images).

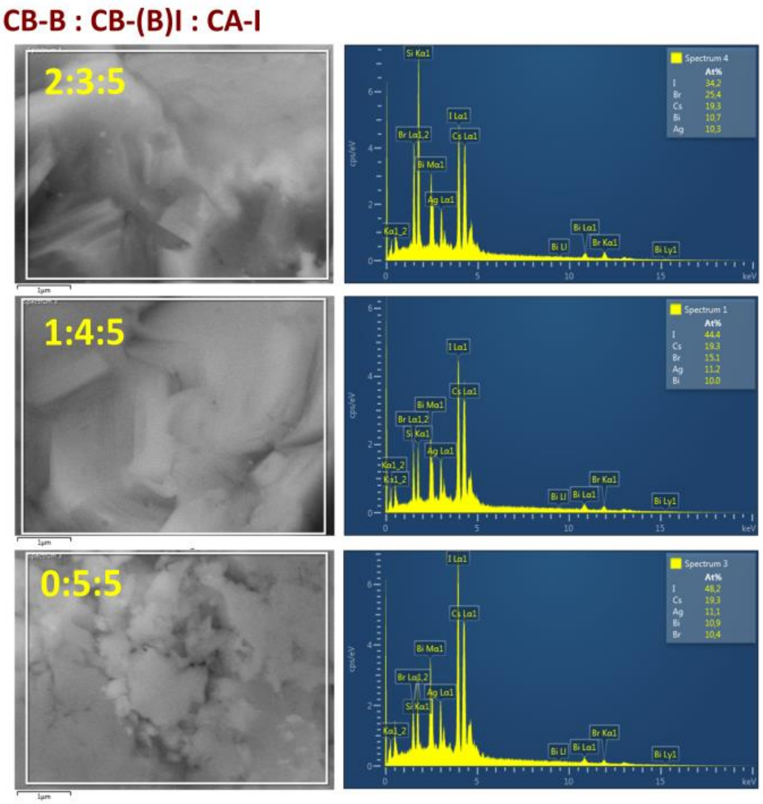

Figure S14. Exemplary SEM images of CAB-(B_yI_{1-y}) perovskites with areas taken for the EDX analysis marked by white rectangles, as well as exemplary EDX spectra of corresponding samples.

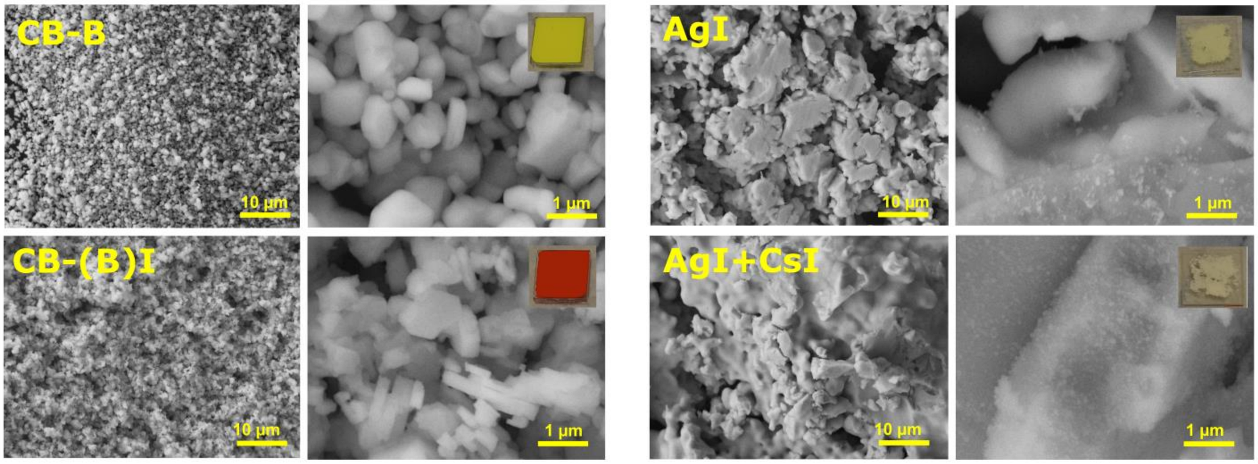

Figure S15. SEM and visual images of CB-B, CB-(B)I, AgI, and AgI+CsI precursors after annealing at 300 ^oC.

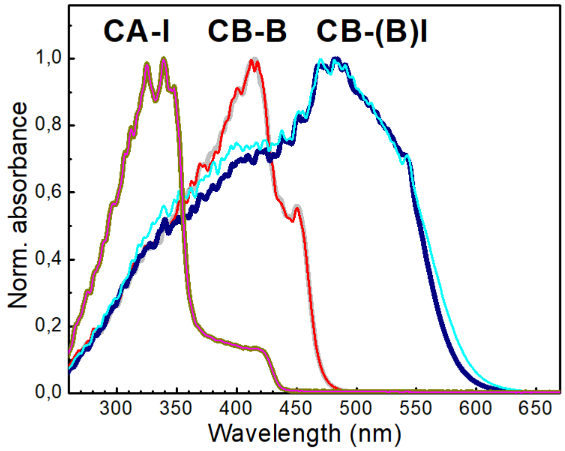

Figure S16. Absorption spectra of CA-I, CB-B, and CB-(B)I double salts before (darker, thicker lines) and after the annealing at 250 °C (brighter, thinner lines).

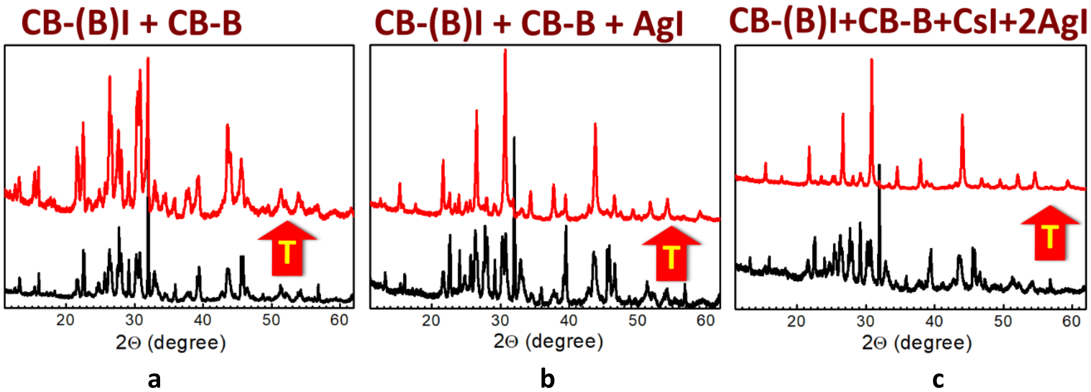

Figure S17. XRD patterns of different combinations of precursors for the synthesis of CAB-(B,I) perovskites before and after annealing at 300 °C.

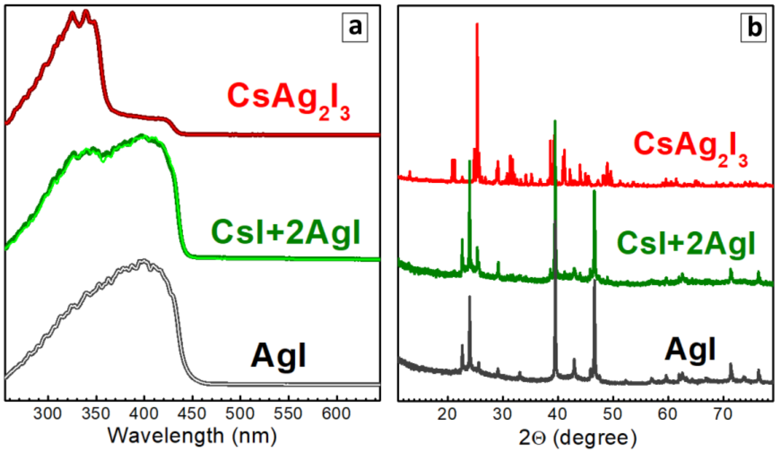

Figure S18. Absorption spectra (a) and powder XRD patterns (b) of AgI, a mixture of CsI and AgI (1:2), and $CsAg_2I_3$ produced by co-precipitation. In (a), thicker, darker lines correspond to non-annealed samples and thinner, brighter lines to the samples annealed at 300 °C. In (b), samples were not annealed.

Tables

Table S1. Parameters of CAB-(B)I perovskites produced at different nominal NaI content at RT AE

Nominal NaI content, %	CAB-(B)I fraction, wt.%	CB-(B)I fraction, wt.%	CA-I fraction, wt.%	$V_{CAB\text{-}(B)I}$, Å^3	Actual iodide fraction in CAB-(B)I, %
100	95	0	5	806	60
120	85	2	13	824	70
133	65	25	10	845	82
140	15	63	22	855	88
150	5	80	15	862	92
160	0	82	18	-	-

Notes: the accuracy of the determination of the CAB-(B)I/CB-(B)I/CA-I fractions, $V_{CAB\text{-}(B)I}$, and actual iodide fraction in CAB-(B)I is 2%, 1 Å^3, and 1%, respectively. The weight fractions of the different phases, as well as the elementary lattice cell volumes, were determined by Rietveld analysis. Accuracies of these determinations were evaluated by performing four independent Rietveld analyses in sequence, averaging the resulting weight fractions, and assessing the average deviation from the mean. The accuracy of iodide content evaluation by EDX was estimated similarly from four independent measurements at different spots on the samples.

Table S2. Mass fractions of CAB-(B)I, CB-(B)I, and CA-I phases and Rietveld refinement goodness parameters R_{wp} and R_{exp} for the series of CAB-(B)I samples produced at different annealing temperatures

Annealing T, (°C)	CAB-(B)I fraction, wt.%	CB-(B)I fraction, wt.%	CA-I fraction, wt.%	R_{wp}, %	R_{exp}, %
20$^{(*)}$	0	77	23	12	9
20	5	75	20	10	8
200	27	55	18	11	9
210	40	43	17	10	9
220	48	36	16	13	9
230	54	33	13	15	9
240	58	33	9	14	9
250	65	27	8	16	9
260	71	24	5	18	9
270	78	20	2	17	10
280	83	15	2	16	9
290	90	9	1	14	9
300	92	7	1	16	10

Notes: $^{(*)}$fitting for two-phase CB-(B)I + CA-I system, without accounting for an admixture of CAB-(B)I

Table S3. Elemental composition, lattice cell volume V, mass fraction *w*, and indirect bandgap E_g of $Cs_2Ag(Bi_xSb_{1-x})(Br_yI_{1-y})_6$ perovskites produced from CB-(B)I+CB-B+CA-I mixtures

***n*(CB-(B)I)**	***x***	***y***	**X/M**	**M/Ag**	**Cs/Bi**	***V* (A^3)**	***w* (%)**	**E_g (eV)**
CB-(B)I+CA-I	1.00	0.17	5.7	1.0	1.9	845.5	80	1.90
CB-(B)I + CS-I + CA-I	0.44	0.08	6.0	1.1	2.0	842.1	85	1.77
CS-I + CA-I	0	<0.01	5.2	1.1	1.8	846.7	95	1.89

Notes: X = Br+I; M = Bi+Sb; elemental ratios are averaged for 4 different measurement spots; the accuracy of the determination of the *V*, *w*, *x*, *y*, and E_g is 1 Å³, 2%, 0.01, 0.01, and 0.01 eV, respectively.

Table S4. Molar fractions *n* of precursor double salts, and lattice cell volume *V*, mass fraction *w*, and indirect bandgap E_g of final $Cs_2AgBi(Br_yI_{1-y})_6$ perovskites produced from CB-(B)I + CB-B + CA-I mixtures

***n*(CB-(B)I)**	***n*(CB-B)**	***n*(CA-I)**	***y***	**X/Bi**	**Bi/Ag**	**Cs/Bi**	***V* (A^3)**	***w* (%)**	**E_g (eV)**
0	5	5	0.76	5.7	1.3	1.9	736.3	92	2.21
1	4	5	0.65	5.8	1.3	2.1	757.1	89	2.15
2	3	5	0.55	5.6	1.1	1.8	780.0	85	2.07
3	2	5	0.42	5.7	1.0	1.8	802.0	92	2.00
4	1	5	0.28	5.9	1.1	1.9	819.6	88	1.95
5	0	5	0.17	5.6	1.0	1.8	846.2	87	1.89

Notes: X = Br+I; M = Bi+Sb; elemental ratios are averaged for 4 different measurement spots; the accuracy of the determination of the *V*, *w*, *y*, and E_g is 1 Å³, 2%, 0.01, and 0.01 eV, respectively.
